# Microbial Ecology of Sheep Milk, Artisanal Feta, and Kefalograviera Cheeses. Part II: Technological, Safety, and Probiotic Attributes of Lactic Acid Bacteria Isolates

**DOI:** 10.3390/foods11030459

**Published:** 2022-02-03

**Authors:** Markella Tsigkrimani, Konstantina Panagiotarea, Spiros Paramithiotis, Loulouda Bosnea, Eleni Pappa, Eleftherios H. Drosinos, Panagiotis N. Skandamis, Marios Mataragas

**Affiliations:** 1 Department of Food Science and Human Nutrition, Agricultural University of Athens, 75 Iera Odos St., 11855 Athens, Greece; marktsigr@aua.gr (M.T.); panagiotarea@gmail.com (K.P.); sdp@aua.gr (S.P.); ehd@aua.gr (E.H.D.); pskan@aua.gr (P.N.S.); 2 Department of Dairy Research, Institute of Technology of Agricultural Products, Hellenic Agricultural Organization “DIMITRA”, 3 Ethnikis Antistaseos St., 45221 Ioannina, Greece; louloudabosnea@gmail.com (L.B.); pappa.eleni@yahoo.gr (E.P.)

**Keywords:** acidification, coagulation, exopolysaccharides, caseinolysis, lipolysis, biogenic amines, hemolysis, antibiotic susceptibility, gelatinase, bile salt hydrolase, survival

## Abstract

The aim of the present study was to examine 189 LAB strains belonging to the species *Enterococcus faecium*, *E. faecalis*, *Lactococcus lactis*, *Pediococcus pentosaceus*, *Leuconostoc mesenteroides*, *Lactiplantibacillus pentosus*, *Latilactobacillus curvatus*, *Lp. plantarum*, *Levilactobacillus brevis*, and *Weissella paramesenteroides* isolated form sheep milk, Feta and Kefalograviera cheeses at different ripening stages, for their technological compatibility with dairy products manufacturing, their activities that may compromise safety of the dairy products as well as their capacity to survive in the human gastrointestinal tract. For that purpose, milk acidification and coagulation capacity, caseinolytic, lipolytic, hemolytic, gelatinolytic, and bile salt hydrolase activity, production of exopolysaccharides, antimicrobial compounds, and biogenic amines, as well as acid and bile salt tolerance and antibiotic susceptibility were examined. The faster acidifying strains were *Lc. lactis* DRD 2658 and *P. pentosaceus* DRD 2657 that reduced the pH value of skim milk, within 6 h to 5.97 and 5.92, respectively. Strains able to perform weak caseinolysis were detected in all species assessed. On the contrary, lipolytic activity, production of exopolysaccharides, amino acid decarboxylation, hemolytic, gelatinase, and bile salt hydrolase activity were not detected. Variable susceptibility to the antibiotics examined was detected among LAB strains. However, in the majority of the cases, resistance was evident. None of the strains assessed, managed to survive to exposure at pH value 1. On the contrary, 25.9 and 88.9% of the strains survived after exposure at pH values 2 and 3, respectively; the reduction of the population was larger in the first case. The strains survived well after exposure to bile salts. The strain-dependent character of the properties examined was verified. Many strains, belonging to different species, have presented very interesting properties; however, further examination is needed before their potential use as starter or adjunct cultures.

## 1. Introduction

The microbiota that drives spontaneous fermentations has been in the epicenter of intensive research for decades. The primary aim was to depict the microecosystem composition, as well as the role, symbiotic patterns, and dynamics of the key players. Product standardization essentially requires the development of starter cultures that provide the necessary metabolic capacities to carry out the desired biotransformations. However, the former are usually accompanied by undesired activities that may compromise the safety of the product. This is also the case of dairy products. The criteria used for the selection of proper starter cultures may be divided into technological, safety, and probiotic-related attributes. 

The most important technological properties include milk acidification, proteolytic and lipolytic activities, as well as production of exopolysaccharides. Milk acidification is very important as it affects microbial stability and enhances clotting and whey expulsion [1]. The proteolytic activities of dairy lactic acid bacteria (LAB) have been extensively studied [2,3]. These affect the organoleptic quality of the final products as well as their functional potential. Indeed, casein hydrolysis may liberate branched-chain, aromatic and sulfur-containing amino acids that through the subsequent transamination and elimination routes may produce several flavor compounds [4]. In addition, the decryption of short peptides with a variety of biological roles, including angiotensin-converting enzyme (ACE) inhibitory, immunomodulatory, antihypertensive, and antithrombotic activities, has also been extensively studied [5]. Free short- and intermediate-chain fatty acids that are liberated from their triglycerides through the activity of lipolytic enzymes are also very important. They may contribute directly to flavor development or serve as precursors for the production of other flavor components such as alcohols, aldehydes, ketones, acids, γ and δ lactones [6]. Exopolysaccharides (EPS) produced by LAB may effectively modify the rheological properties and concomitantly the mouthfeel of the products [7]. In addition, health promoting activity has been claimed for some of them [8,9,10].

Several metabolic activities of dairy microbiota may improve or compromise safety of the final product; these include production of antimicrobial compounds, hemolytic, gelatinase, and amino acid decarboxylase activities, as well as antibiotic susceptibility. The latter is very useful as it may indicate presence of antibiotic resistance genes. Indeed, LAB are considered as a pool of transferable antibiotic resistance genes, the spread of which has been recognized as a major problem for public health [11,12]. Production of antimicrobial compounds is a useful trait that may assist in controlling of foodborne pathogens occurrence and proliferation. Indeed, the capability of LAB to produce an extended variety of antimicrobial compounds is well established. Among them, production and application of proteinaceous antimicrobial compounds in food biopreservation have drawn specific attention over the last decades [13,14]. Hemolytic and gelatinase activities contribute to virulence of enterococci. In both cases, assessment of the phenotype is necessary since predictive accuracy of genetic determinants is still low [15,16]. Decarboxylation of amino acids to their respective amines is an attempt to cytosol deacidification, which results from growth under acidic conditions [17]. Accumulation of biogenic amines is not desired since their consumption has been correlated with a series of adverse effects, including headache, skin irritation, etc. [18].

The probiotic-related properties are constantly updated, mostly with ones related to the functional potential of the cultures. However, survival in the gastrointestinal tract (GIT) and production of bile salt hydrolase are among the key characteristics that a potential probiotic strain may possess [19].

The aim of the present study was to assess the technological, safety, and probiotic properties of lactic acid bacteria isolates from milk and dairy products. More specifically, 189 isolates belonging to the different genera, i.e., *Enterococcus*, *Lactococcus*, *Pediococcus*, *Leuconostoc*, *Lactiplantibacillus*, *Latilactobacillus*, *Levilactobacillus,* and *Weissella* were examined for their milk acidification and coagulation capacity, caseinolytic, lipolytic, hemolytic, gelatinolytic, and bile salt hydrolase activity, production of exopolysaccharides, antimicrobial compounds, and biogenic amines, as well as acid and bile salt tolerance and antibiotic susceptibility.

## 2. Materials and Methods

### 2.1. Microbial Strains and Culture Conditions

A total of 189 lactic acid bacteria (LAB) strains of the collection of Dairy Research Department (DRD) of Hellenic Agricultural Organization “DIMITRA” (ELGO-DIMITRA), isolated from sheep milk and artisanal Feta and Kefalograviera cheeses [20], belonging to the species *Enterococcus faecium* (46 strains), *E. faecalis* (3 strains), *Lactococcus lactis* (7 strains), *Pediococcus pentosaceus* (29 strains), *Leuconostoc mesenteroides* (6 strains), *Lactiplantibacillus pentosus* (2 strains), *Latilactobacillus curvatus* (6 strains), *Lp. plantarum* (29 strains), *Levilactobacillus brevis* (29 strains), and *Weissella paramesenteroides* (32 strains) were included in the present study (Table S1). All strains were stored at −20 °C, in Nutrient Broth (LAB M, Lancashire, UK) supplemented with 30% glycerol (Applichem, Darmstadt, Germany). Before experimental use, strains were subcultured twice in de Mann, Rogosa, and Sharpe (MRS) broth (LAB M) at 37 °C for enterococci and 30 °C for the rest LAB, for 24 h. Purity was examined after streaking in MRS agar. Before inoculation, the bacterial cultures were washed twice with sterile saline and resuspended in the same diluent at the initial culture volume, unless otherwise stated. 

### 2.2. Technological Properties

#### 2.2.1. Milk Acidification and Coagulation

Milk acidification and coagulation was examined in 74 LAB representative strains, which were selected based on their different isolation source or separate genotypic cluster [20], namely *E. faecium* (strains DRD 2579, 2581, 2585, 2587, 2588, 2600, 2602, 2667, 2670, 2686, 2691, 2695, 2711, 2720, 2723, 2724), *Lv. brevis* (strains DRD 2596, 2624, 2664, 2665, 2688, 2757, 2760, 2763), *Lp. plantarum* (strains DRD 2584, 2608, 2612, 2645, 2648, 2653, 2656, 2668), *E. faecalis* (strains DRD 2602, 2651, 2722), *Lp. pentosus* (strain DRD 2662, 2693), *Lc. lactis* (strain DRD 2646, 2658, 2692, 2699), *Lt. curvatus* (strains DRD 2708, 2709, 2716, 2728), *W. paramesenteroides* (strains DRD 2613, 2701, 2714, 2719, 2726, 2743, 2751, 2755, 2756), *Ln. mesenteroides* (strains DRD 2576, 2604, 2703, 2705, 2706), and *P. pentosaceus* (strains DRD 2616, 2634, 2635, 2652, 2655, 2657, 2676, 2681, 2689, 2694, 2748, 2749, 2752, 2753, 2761). Overnight bacterial cultures of the representative strains were used to inoculate (1% inoculum) 10 mL skim milk (LAB M). Incubation took place at 37 °C for enterococci and 30 °C for the rest LAB, for 24 h. The pH value was measured immediately upon inoculation and for every 3 h until 24 h. After each measurement, the respective test tube was discarded. The ΔpH values presented were calculated by subtracting the pH value after the given incubation time at 30 °C (37 °C for enterococci) from the respective of uninoculated skim milk incubated for the same time at the same temperature.

Milk coagulation was performed according to Bancalari et al. [21]. In brief, overnight cultures of the aforementioned strains were inoculated (2% inoculum) into 10 mL skim milk (LAB M) and incubated at 37 °C for enterococci and 30 °C for the rest of the LAB, for up to 7 days. Milk coagulation was optically evaluated at 6, 12, 24 h, 2, 3, 4, 5, 6 and 7 days. The time in which coagulation was visibly observed in presented.

#### 2.2.2. Caseinolytic and Lipolytic Capacity

The caseinolytic and lipolytic capacity of all strains were assessed through agar well diffusion assays. In the first case, wells were aseptically punched in freshly prepared lawns of a medium consisting of 0.5% tryptone (LAB M), 0.25% yeast extract (LAB M), 0.1% glucose (Applichem), 1% casein (Sigma-Aldrich, St. Louis, MO, USA), and 1.5% bacteriological agar (LAB M), pH 6.9. Overnight bacterial cultures were centrifuged (12,000× *g*; 15 min; 4 °C) and 25 μL of each cell-free supernatant was added in each well. The plates were incubated at 30 °C for 5 days. The plates were then stained with 0.05% (*w*/*v*) Coomassie Brilliant Blue G-250 (Applichem). Presence of a clarification halo around each well was perceived as an indication of caseinolytic activity. The latter was characterized as weak, if the clarification zone extended for less than 0.5 cm around the wells, and strong if the clarification zone extended for more than 0.5 cm. For the assessment of the lipolytic capacity, wells were aseptically punched in freshly prepared lawns of a medium consisting of 0.5% peptone (LAB M), 0.3% meat extract (LAB M), 0.5% lecithin (Serva, Heidelberg, Germany), 1% tributyrin (Merck, Darmstadt, Germany), 3% meat extract (Merch), and 1.5% bacteriological agar (LAB M), with the addition of 2.5 mM CaCl_2_ (Applichem) and 5 mM MgSO_4_ (Applichem) [22]. Overnight bacterial cultures were centrifuged (12,000× *g*; 15 min; 4 °C) and 25 μL of each cell-free supernatant was added in each well. The plates were incubated at 30 °C for 10 days. Presence of a clarification halo around each well was perceived as an indication of lipolytic activity. As in the case of proteolytic activity, it was characterized as weak, if the clarification zone extended for less than 0.5 cm around the wells, and strong if the clarification zone extended for more than 0.5 cm. 

#### 2.2.3. Production of Exopolysaccharides (EPS)

The capacity of all strains to produce exopolysaccharides was assessed according to Smitinont et al. [23]. In brief, overnight bacterial cultures were streaked on the surface of MRS agar (LAB M) containing 2% glucose, galactose, or lactose (Applichem). Occurrence of slimy phenotype after incubation at 37 °C for enterococci and 30 °C for the rest LAB for 3 days was indicative of EPS production. 

### 2.3. Safety Properties

#### 2.3.1. Production of Antimicrobial Compounds

Production of antibacterial compounds against a mixture of five strains from each of *Listeria monocytogenes*, *Salmonella enterica,* and *Escherichia coli* O157:H7 pathogen was assessed through agar well diffusion assay. More accurately, wells were aseptically punched in freshly prepared BHI agar (LAB M) lawns inoculated with the mixture of the pathogens. Overnight bacterial cultures of all strains were centrifuged (12,000× *g*; 15 min; 4 °C), each cell-free supernatant was neutralized and treated with catalase (Sigma-Aldrich) and 25 μL of each was added in each well. The plates were incubated at 37 °C for 24 h. Growth inhibition of the pathogenic bacteria strain mixture used as indicator around the wells was indicative of the presence of antibacterial compounds in the respective cell-free supernatant. The protein nature of the antibacterial compounds was verified by treatment with proteinase (Sigma-Aldrich) and trypsin (Sigma-Aldrich), according to Syrokou et al. [24]. 

#### 2.3.2. Biogenic Amine Production

The capacity of all bacterial strains to decarboxylate lysine (Serva), tyrosine (Serva), ornithine (Applichem), and histidine (Applichem) was assessed by using the substrate described by Bover-Cid and Holzapfel [25]. Overnight bacterial cultures were used to inoculate the aforementioned substrate supplemented with each amino acid. Incubation took place at 37 °C for enterococci and 30 °C for the rest of the LAB for 48 h; occurrence of color change around the colonies was indicative of the respective amino acid decarboxylation. 

#### 2.3.3. Hemolytic Activity

Hemolytic activity was assessed with the use of Columbia blood agar (Oxoid, Hampshire, UK) supplemented with 5% defibrinized horse blood (TCS Biosciences, Buckingham, UK). Overnight bacterial cultures of all strains were used to inoculate the aforementioned substrate. Examination of the plates for α-hemolysis (green discoloration around the colonies), β-hemolysis (clarification around the colonies), or γ-hemolysis (no discoloration or clarification) took place after incubation at 37 °C for enterococci and 30 °C for the rest LAB, for 48 h.

#### 2.3.4. Antibiotic Susceptibility

The resistance of all strains to eight antibiotics, namely ampicillin (Applichem), bacitracin (Sigma), chloramphenicol (Applichem), erythromycin (Sigma), novobiocin (Sigma), streptomycin (Applichem), tetracycline (Applichem), and vancomycin (Applichem) was estimated using the Kirby-Bauer disk diffusion susceptibility test [26]. In brief, 6-mm filter paper disks were impregnated with known concentration of the aforementioned antibiotics (0, 1, 2, 4, 8, 16, 32, 64, and 128 mg/mL) and placed on the surface of an MRS agar plate, on the surface of which 100 μL of 10^7^ colony forming units per mL (CFU/mL) dilution of each strain included in the present study, was spread. Growth of the strains around the disks was indicative of their resistance to the respective concentration.

#### 2.3.5. Gelatinase Activity

Gelatinase activity was assessed by the nutrient gelatin stab method and the nutrient gelatin plate method according to Cruz and Torres [27]. In brief, overnight bacterial cultures of all strains were used to inoculate both substrates. Incubation took place at 25 °C for 1 week in the first case and at 35 °C for 24 h in the latter. After incubation, the substrates were examined for liquefaction in the first case and clear zones around the colonies in the latter. 

### 2.4. Probiotic-Related Activities

#### 2.4.1. Acid and Bile Salt Tolerance

Acid and bile salt tolerance of all strains was assessed according to Paramithiotis et al. [28]. More accurately, overnight bacterial cultures were washed twice with sterile saline. Acid tolerance was assessed after resuspension of the biomass in phosphate-buffered saline (PBS) buffer adjusted to pH 1, 2, or 3 with HCl and incubation at 37 °C for 3 h. Bile salt tolerance was assessed after resuspension of the biomass in PBS buffer, pH 8, containing 0.5, 1.0, or 2.0% (*w*/*v*) bile bovine (Sigma) and incubation at 37 °C for 4 h. In both cases, the viable cell counts enumerated immediately upon inoculation and after the incubation period were used to estimate acid and bile salt tolerance. If the surviving population was below the enumeration limit (10 CFU/mL), the latter was used for calculating purposes.

#### 2.4.2. Bile Salt Hydrolase Activity

Bile salt hydrolase activity was performed according to Paramithiotis et al. [28]. In brief, overnight bacterial cultures of all strains were streaked on MRS agar, supplemented with 0.5% (*w*/*v*) bile bovine, and incubated at 37 °C for 48 h. Bile bovine hydrolysis was indicated by precipitation zones around the colonies and altered colony morphology.

## 3. Results

### 3.1. Technological Properties

#### 3.1.1. Milk Acidification and Coagulation

In Table 1, skim milk acidification (in ΔpH) and coagulation capacity of representative strains of the LAB included in the present study, is presented. In general, skim milk acidification and coagulation seemed to be a strain- rather than a species-dependent property. Strains with variable acidification and coagulation capacity were included in all species. *Enterococcus faecalis* was an exception, since coagulation was achieved after 24 h, by all strains. The strains that achieved faster acidification were *Lc. lactis* DRD 2658 and *P. pentosaceus* DRD 2657. In both cases, coagulation of skim milk was observed after 6 h. Fast acidification and coagulation (after 6 h) was also achieved by *Lc. lactis* DRD 2692, *Lp. pentosus* DRD 2662, *P. pentosaceus* DRD 2655, and *Lv. brevis* DRD 2664.

#### 3.1.2. Caseinolytic and Lipolytic Capacity

Weak caseinolytic activity was observed for 64 LAB strains belonging to *E. faecium* (9 strains; DRD 2577, 2581, 2582, 2586, 2588, 2636, 2718, 2721, and 2725), *Ln. mesenteroides* (5 strains; DRD 2576, 2604, 2703, 2704, and 2705), *Lp. plantarum* (15 strains; DRD 2606, 2607, 2608, 2609, 2610, 2611, 2612, 2614, 2615, 2617, 2618, 2619, 2637, 2640, and 2646), *P. pentosaceus* (11 strains; DRD 2616, 2621, 2622, 2623, 2683, 2696, 2748, 2749, 2752, 2753, and 2761), *W. paramesenteroides* (16 strains; DRD 2613, 2712, 2713, 2714, 2719, 2740, 2741, 2742, 2743, 2744, 2745, 2746, 2747, 2750, 2755, and 2756), *Lv. brevis* (3 strains; DRD 2684, 2685, and 2759), *Lc. lactis* (1 strain; DRD 2697), *Lt. curvatus* (2 strains; DRD 2708 and 2716), *Lp. pentosus* (1 strain; DRD 2693) and *E. faecalis* (1 strain; DRD 2722). Since the caseinolytic activity was weak, no further assessment with SDS-PAGE took place. As far as lipolytic activity was concerned, this was not observed by the strains under study.

#### 3.1.3. Production of Exopolysaccharides (EPS)

None of the strains under study presented the slimy phenotype when glucose, galactose, or lactose were supplied as carbon sources.

### 3.2. Safety Properties

#### 3.2.1. Production of Antimicrobial Compounds

A total of 14 strains produced antimicrobial compounds against the *L. monocytogenes* strain mixture that was employed as indicator. These belonged to *E. faecium* (10 strains; strains DRD 2577, 2579, 2580, 2581, 2582, 2585, 2588, 2686, 2721, and 2723), *Lp. plantarum* (1 strain; DRD 2650), *Lv. brevis* (1 strain; DRD 2629), and *Ln. mesenteroides* (2 strains; DRD 2576 and 2705). The protein nature of these compounds was verified after treatment with proteolytic enzymes; in all cases the antilisterial activity was abolished. None of the strains produced antimicrobial compounds against *E. coli* O157:H7 or *S. enterica* strain mixtures.

#### 3.2.2. Biogenic Amine Production

None of the strains under study presented with the necessary color change that was indicative of decarboxylation of lysine, tyrosine, ornithine, or histidine that were provided in the growth medium.

#### 3.2.3. Hemolytic Activity

None of the strains under study presented hemolytic activity and thus can be classified as non-hemolytic or γ-hemolytic.

#### 3.2.4. Antibiotic Susceptibility

**Figure 1:**
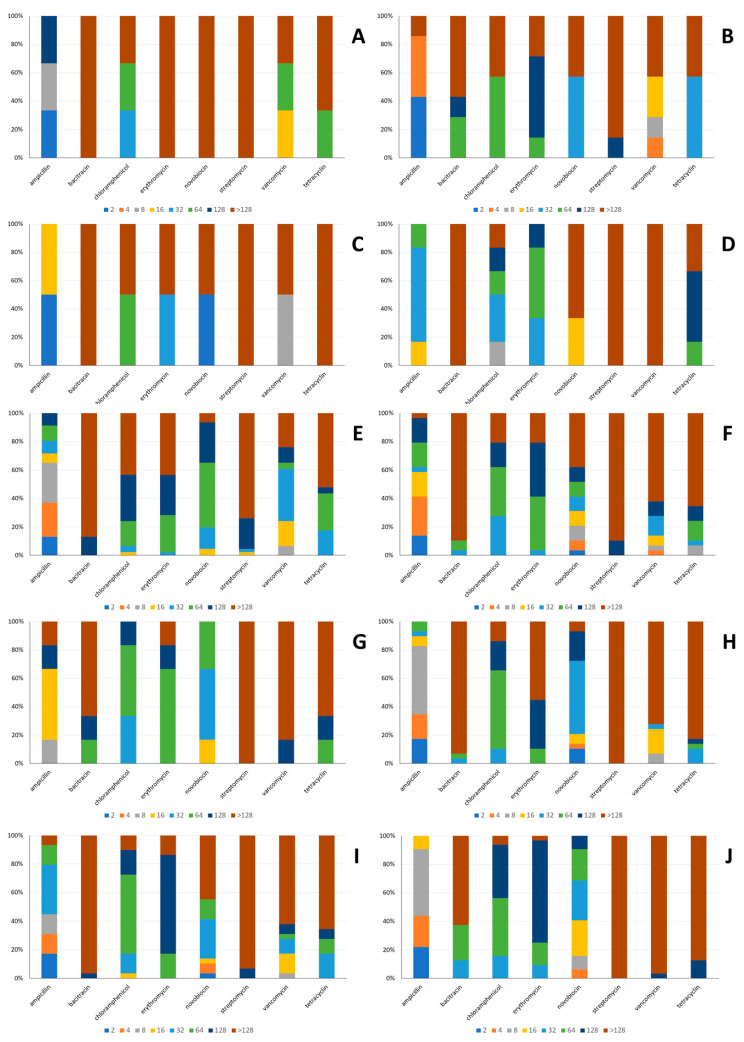
Antibiotic susceptibility of *E. faecalis* (**A**), *Lc. lactis* (**B**), *Lp. pentosus* (**C**), *Lt. curvatus* (**D**), *E. faecium* (**E**), *P. pentosaceus* (**F**), *Ln. mesenteroides* (**G**), *Lp. plantarum* (**H**), *Lv. brevis* (**I**), and *W. paramesenteroides* (**J**). The concentration of each antibiotic that was necessary to inhibit growth of the strains of each species included in the present study (in mg/mL), indicated in colors.

The antibiotic susceptibility of the strains under study is presented in Figure 1A–J. A variable degree of susceptibility to the antibiotics examined was observed, with the exception of streptomycin and bacitracin, the concentration of which required to inhibit growth of the majority of the strains under study was above 128 mg/mL. Tetracycline and erythromycin were also relatively ineffective since the concentration required to inhibit growth of the majority of the strains under study was equal or above 64 mg/mL, with the exception of *Lc. lactis* and *Lp. pentosus*. In the first case, growth of the majority of the strains was inhibited by 32 mg/mL tetracycline, whereas growth of half of the *Lp. pentosus* was inhibited by 32 mg/mL and the other half by more than 128 mg/mL erythromycin. Regarding novobiocin, growth of the majority of the strains belonging to *E. faecalis*, *Lp. pentosus*, *Lt. curvatus*, *E. faecium*, *P. pentosaceus,* and *Lv. brevis* was inhibited by concentration equal or above 64 mg/mL. On the contrary, the strains belonging to *Lc. lactis*, *Ln. mesenteroides*, *Lp. plantarum,* and *W. paramesenteroides* seemed to be more susceptible to novobiocin since a concentration equal or less than 32 mg/mL was required to inhibit growth of the majority of them. As far as vancomycin was concerned, growth of the majority of the strains belonging to *E. faecalis*, *Lt. curvatus*, *P. pentosaceus*, *Ln. mesenteroides*, *Lp. plantarum*, *Lv. brevis,* and *W. paramesenteroides* was inhibited by concentration equal or above 64 mg/mL. On the contrary, half of the *Lp. pentosus* strains were inhibited by 8 mg/mL and the other half by more than 128 mg/mL, whereas growth of the majority of *Lc. lactis* and *E. faecium* strains was inhibited by concentration equal or less than 16 and 32 mg/mL, respectively. Finally, ampicillin seemed to be the most effective among the antibiotics examined. Growth of the majority of *Lt. curvatus* and *Lv. brevis* strains was inhibited by concentration equal or less than 32 mg/mL, *Lp. pentosus*, *P. pentosaceus* and *Ln. mesenteroides* equal or less than 16 mg/mL, *E. faecalis*, *E. faecium*, *Lp. plantarum* and *W. paramesenteroides* equal or less than 8 mb/mL and *Lc. lactis* equal or less than 4 mg/mL.

#### 3.2.5. Gelatinase Activity

None of the strains under study presented gelatinase activity, by any of the methods employed.

### 3.3. Probiotic-Related Activities

#### 3.3.1. Acid and Bile Salt Tolerance

**Figure 2:**
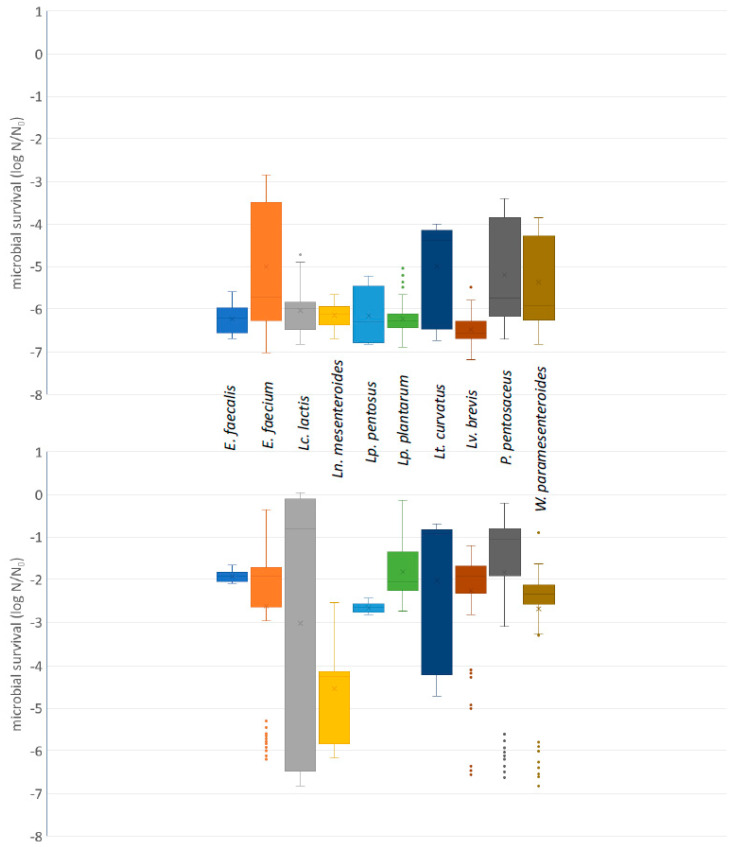
Box and whisker plot, summarizing the microbial survival (in log N/N_0_) of the strains included in the present study, at species level after exposure to PBS buffer adjusted to pH 2 (**upper**) or 3 (**lower**) with HCl and incubation at 37 °C for 3 h. N: microbial population after incubation; N_0_: initial microbial population; solid circle symbol: outliers.

In Figure S1, the microbial survival after exposure of the strains to PBS buffer adjusted at pH values 2 and 3 and incubation at 37 °C for 3 h, is presented. A box and whisker plot, summarizing the survival of the strains, at species level, is shown in Figure 2. After exposure to PBS buffer adjusted at pH value 1 and incubation at 37 °C for 3 h, no surviving population was enumerated; therefore, these results were not included in Figure 2 and Figure S1. Exposure to PBS buffer adjusted at pH values 2 and 3 and incubation at 37 °C for 3 h resulted in reduction of the population of all strains. In the former case, the reduction of the population was larger (*p* < 0.05) in all strains, with the exception of the ones in which no surviving population was detected in both pH values. 

The median values of the microbial survival after exposure to pH 2 ranged from −6.56 log N/N_0_ in *Lv. brevis* to −4.38 log N/N_0_ in *Lt. curvatus* and after exposure to pH 3 from −4.24 log N/N_0_ in *Ln. mesenteroides* to −0.81 log N/N_0_ in *Lc. lactis*.

**Figure 3:**
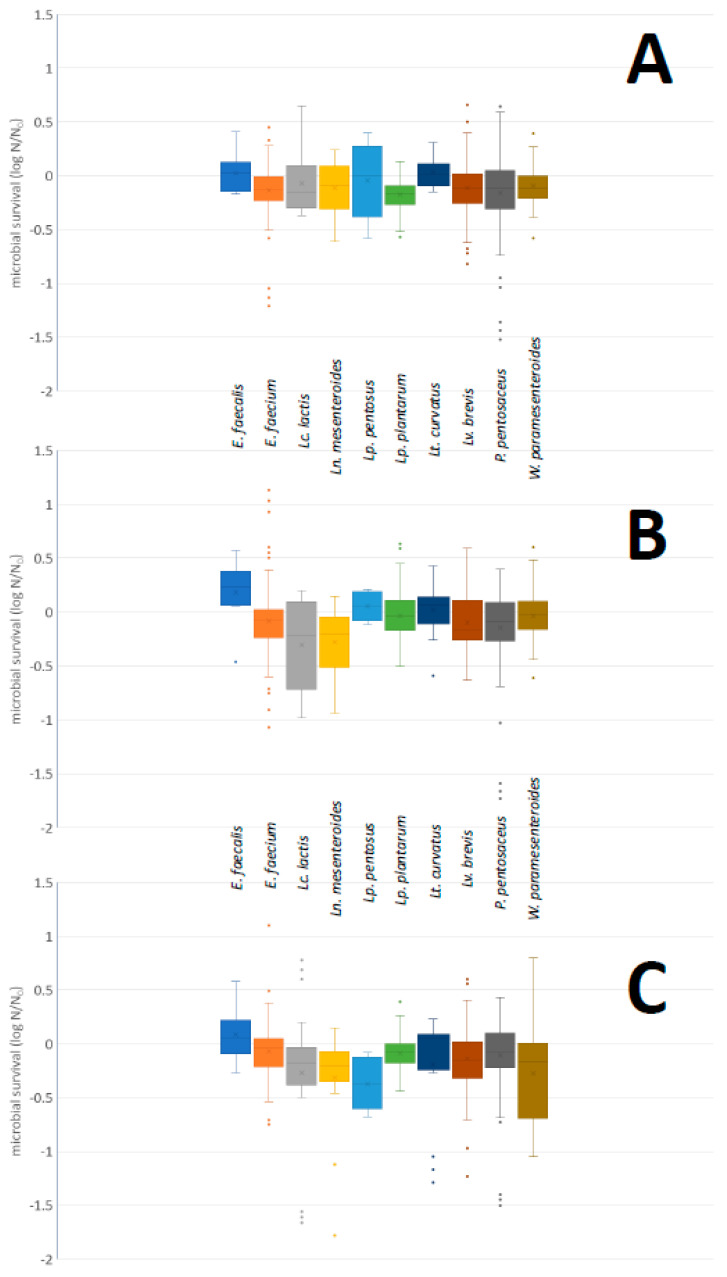
Box and whisker plot, summarizing the microbial survival (in log N/N_0_) of the strains included in the present study, at species level after exposure to PBS buffer, pH 8, containing 0.5% (**A**), 1.0% (**B**), or 2.0% (**C**) (*w*/*v*) bile bovine and incubation at 37 °C for 4 h. N: microbial population after incubation; N_0_: initial microbial population; solid circle symbol: outliers.

Bile tolerance of the strains included in the present study is presented in Figure S2. A box and whisker plot, summarizing the survival of the strains after incubation, at species level, is shown in Figure 3. The lowest survival at 0.5 and 1.0% referred to *P. pentosaceus* strain DRD 2761, while at 2% bile bovine concentration to *Ln. mesenteroides* strain DRD 2604. Growth was also observed in many cases; it reached 0.66, 1.13, and 1.10 log N/N_0_ at 0.5, 1.0, and 2.0% bile bovine, respectively. In the first case, such a growth was observed in the case of *Lv. brevis* strain DRD 2632, in the second case in *E. faecium* strain DRD 2723 and in the third case in *E. faecium* strain 2667. The median values of the microbial survival ranged from −0.17 to 0.03, from −0.22 to 0.23, and from −0.37 to 0.06 log N/N_0_ at 0.5, 1.0 and 2.0% bile bovine, respectively. The lowest median values were observed for *Lp. plantarum* at 0.5% bile bovine, for *Lc. lactis* at 1.0%, and for *Lp. pentosus* at 2.0% bile bovine. On the other hand, the highest median values were observed in all cases for *E. faecalis*.

#### 3.3.2. Bile Salt Hydrolase Activity

None of the strains under study presented with the necessary precipitation zones around the colonies or the altered colony morphology, when grown on the surface of MRS agar, which was indicative of bile salt hydrolase activity.

## 4. Discussion

In recent years, consumer awareness regarding food safety and nutritional status is increasing. In addition, the demand for functional food, i.e., food that may provide more than just nutritional elements [29] is also increasing. Dairy industry is addressing these demands through the selection of microorganisms that may serve as starter or adjunct cultures that apart from the product-dependent sensorial and technological characteristics, may provide with additional benefits. In the present study, the technological, safety and probiotic properties of lactic acid bacteria isolated from sheep milk and during ripening of Feta and Kefalograviera cheeses are assessed. To our knowledge, there is a knowledge gap, especially regarding the properties of Kefalograviera wild microbiota.

Milk acidification and coagulation, caseinolytic and lipolytic activity, as well as production of exopolysaccharides are among the critical technological properties for LAB that are evaluated as starter or adjunct cultures in cheesemaking. The rapid pH reduction contributes to safety, shaping of appropriate environment for lipolytic, and proteolytic enzymes and it is essential for milk coagulation [30,31]. Caseinolytic and lipolytic activity as well as EPS production play a major role in aroma, flavor, and texture development of final products [32,33]. 

Rapid milk acidification is one of the most important properties of LAB that are used as starter cultures, but it is not desirable in the adjunct cultures, since it could negatively affect the quality of the final products [34]. Therefore, strains with poor acidifying activity but with interesting technological or functional properties, can be used as adjunct cultures and combined with more acidifying strains, in mixed starter cultures [30,35]. According to Beresford et al. [36], the starter bacteria should reduce the pH value of milk to 5.3 within 6 h at 30–37 °C. In the present study, the faster acidifying strains were *Lc. lactis* DRD 2658 and *P. pentosaceus* DRD 2657, which reduced the pH value of skim milk, within 6 h, to 5.97 and 5.92, respectively, failing to meet this criterion. The results obtained in the present study highlighted the strain-dependent character of this property. Indeed, in all species, faster and slower acidifying strains were included, concurring with the results presented in the literature [30,31,32,33,34,37,38,39]. The only exception was *E. faecalis*, in which a uniformity was observed in the acidifying capacity, which may be assigned to the small number of strains assessed. Milk coagulation is encouraged by acid production from LAB strains, as well as coagulant enzymes activity [36,38]. This property is also strain-dependent and strains able to coagulate skim milk within 24 h were detected in all species included in the present study. Similar results were also presented by Fusieger et al. [37], Teixeira et al. [39], and Picon et al. [40].

Proteolytic LAB strains, through the activity of cell wall-associated proteinases and intracellular peptidases, degrade casein leading to the development of aroma, texture, and flavor [37,41,42]. Therefore, caseinolysis is of great importance for starter or adjunct LAB. Nevertheless, extensive caseinolysis could result in organoleptic and structural defects [37,43]. In the present study, weak caseinolytic strains were detected in all species assessed. Interestingly, the majority of them were isolated from the same source, namely Feta cheese in early ripening stage [20]. Caseinolysis does not seem to be a property shared among the majority of dairy LAB; in addition, the extend of proteolytic activity is strain-dependent [30,32,34,37,38,39,44,45,46,47,48].

Lipolytic activity is another important trait that may affect cheese texture, aroma, and flavor [30,49,50]. In the present study, no lipolytic strains were detected, supporting the conclusion that lipolysis is rather uncommon among dairy LAB [30,31,32,33,38,43,46,48,49,50,51].

Exopolysaccharides production by LAB used in cheese-making is important from both a technological and functional perspective. More specifically, these molecules may function as thickeners and stabilizers, improving texture and structure of products. Moreover, EPS may have a positive effect on consumer health [48,50,52]. In the present study, no EPS producing strain was detected, concurring with Camara et al. [46], who characterized this property as rare among dairy LAB. However, EPS producing strains have been isolated from milk and dairy products [35,48,50,52,53].

Regarding the safety traits that were assessed in the present study, none of the isolates exhibited amino acid decarboxylation, hemolytic and gelatinase activity, while their response to antibiotic varied widely; finally, 14 out of 189 strains were found to produce antibacterial compounds able to inhibit growth of the *L. monocytogenes* strain mixture that was used as indicator.

LAB may decarboxylate amino acids and produce biogenic amines during the production and storage of fermented products. The consumption of biogenic amines may lead to series of adverse health effects [18]. Biogenic amines production seems to be a rather common property among dairy LAB [40,46,48,50,53]. However, in the present study no such strains were detected. This could be attributed to the method used for their detection and should be verified by analytical techniques with improved limit of detection, such as High-Performance Liquid Chromatography (HPLC). 

Hemolytic and gelatinase activities are also very important; strains possessing such activities constitute a hazard to consumers health [45,52,54,55]. In general, no such activities have been reported for dairy LAB with the exception of enterococci, in which occurrence of hemolytic and gelatinolytic strains has been reported [33,48,52,56,57]. No such strains were detected in the present study.

The bacterial antibiotic resistance is a matter of great concern for the food industry. The phenotypical assessment of LAB antibiotic susceptibility is the first step to evaluate the presence of antibiotic resistance genes [32]. The majority of the strains included in the present study, exhibited resistance to the antibiotics examined. According to the cut-off values recommended by EFSA [58], all isolates, with only a few exceptions, seemed to be resistant to chloramphenicol, erythromycin, streptomycin, and tetracycline. Similar was the case of ampicillin, but more strains could be characterized as sensitive. Regarding vancomycin, all *E. faecalis*, *E. faecium*, *Lc. lactis,* and *W. paramesenteroides* strains, can be characterized as resistant. For the remaining species, no cut-off values have been proposed for vancomycin.

According to the Clinical and Laboratory Standards Institute (CLSI) [59,60], all *E. faecium*, *E. faecalis*, *Lt. curvatus*, *Lp. pentosus*, *Lp. plantarum*, *Lc. lactis,* and *Lv. brevis* strains can be characterized as resistant to erythromycin. Similarly, all *Enterococcus* spp. and *Lc. lactis* strains to tetracycline. Moreover, none of the *Enterococcus* spp., *Ln. mesenteroides,* and *P. pentosaceus* strains appeared to be sensitive to chloramphenicol, except one *E. faecium* isolate (DRD 2724). Regarding ampicillin, the results varied between species and strains; the higher rate of ampicillin susceptible strains was observed for *Lp. plantarum* (82.8%), followed by *E. faecalis* (66.7%) and *E. faecium* (65.2%), while none of *Lt. curvatus* as well as *Lc. lactis* seemed to be ampicillin sensitive. *Enterococcus faecalis*, *Lt. curvatus*, *Lc. lactis*, one out of two *Lp. pentosus,* as well as the majority of *E. faecium*, *Lp. plantarum,* and *Lv. brevis* strains seemed to be resistant to vancomycin. Regarding novobiocin, strains belonging to *Ln. mesenteroides*, *Lc. lactis*, *Lp. plantarum,* and *W. paramesenteroides* seemed to be more susceptible than *E. faecium*, *E. faecalis*, *Lt. curvatus*, *Lp. pentosus*, *Lv. brevis,* and *P. pentosaceus*. 

In general, *Enterococcus* spp. exhibit natural resistance to many antibiotics, such as ampicillin, streptomycin, tetracyclines, chloramphenicol, and vancomycin [33,42], while *Lactococcus* spp. and *Pediococcus* spp. present intrinsic resistance to vancomycin and tetracycline [61]. However, in many studies, susceptibility of dairy LAB has been exhibited [32,47,56,62,63]. 

In recent years, the capacity of LAB as natural biopreservatives, especially in fermented foods, has been exhibited. This capacity is attributed to the production of various metabolites with antimicrobial activity, such as organic acids (lactic, acetic, propionic, and formic acids), hydrogen peroxide, diacetyl, carbon dioxide, ethanol, and bacteriocins. The latter are ribosomally synthesized proteinaceous molecules that target mainly the bacterial cytoplasmic membrane [33,39,52]. *L. monocytogenes*, *E. coli,* and *Salmonella* are among the most frequent foodborne pathogens and the antimicrobial potential of LAB against them has been extensively assessed. In the present study, 14 strains exhibited such activity against the *L. monocytogenes* strains mixture. Noticeably, ten of those strains belonged to the genetically closely related *E. faecium*, which explains this activity [64]. In line with present data, Albayrak and Duran [32] reported that the majority of *Enterococcus* spp. isolated from a ripened white cheese sample, inhibited growth of *L. monocytogenes*. In addition, Ispirli et al. [65] reported that 12 *Enterococcus* spp., isolated from Turkish white cheese, produced bacteriocins against Gram-negative bacteria, such as *S*. Typhimurium and *E. coli*. In the present study, one *Lp. plantarum*, one *Lv. brevis,* and two *Ln. mesenteroides* strains also exhibited antilisterial activity. Such activity from strains belonging to these species is commonly reported in literature [33,52,56].

Regarding the probiotic potential of LAB isolates, acid and bile tolerance, as well as bile salt hydrolase (BSH) activity were tested since resistance to gastric acidity and bile salts is essential for the survival of microorganisms in the gastrointestinal tract (GIT). The capacity of LAB to survive acidic conditions is essential, since in the stomach, they may confront pH values less than 3.0 [47]. In the present study, none of the 189 LAB strains assessed, managed to survive to exposure at pH value 1. On the contrary, 25.9 and 88.9% of the strains survived after exposure at pH values 2 and 3, respectively. The population reduction was greater in the first case. Similarly, tolerance to exposure at pH 3.0, but population reduction greater than 2 log CFU/mL of 116 LAB strains isolated from Serpa cheese was reported by Ruiz-Moyano et al. [63]. Zoumpopoulou et al. [56] reported that 32 isolates belong to 14 different species, isolated from Kalathaki and Melichloro cheeses presented with population reduction larger than 2 log CFU/mL after exposure to pH 2.5 for 2 h. Survival rates ranging from 70–90% were reported by Islam et al. [48] when 11 strains belonging to different species were exposed to pH 2 for 3 h. 

The transit time of LAB in human GIT is estimated from 4 to 6h, and the bile concentration from 0.3 to 0.5% w/v, depending on the person [32,49]. In the present study, the maximum population reduction observed was 1.5 log CFU/mL, in any bile salt concentration studied. In some cases, growth enhancement was observed. Similarly, Albayrak and Duran [32] observed enhancement of *Enterococcus* spp. growth, the population increase of which, in some cases exceeded 1 log CFU/mL. In general, good survival of dairy LAB is reported [47,56], with just a few exceptions [34]. 

Bile salts are antimicrobial agents that interact with the cell membrane, proteins, DNA, and chelate iron and calcium. The deconjugation of bile salts from BSH enzyme contribute to bacterial survival in the GIT and has an important role in intestinal homeostasis and in the reduction of serum cholesterol levels. Therefore, BSH activity is a desirable property. In the present study, no such strains were detected. This concurs with the conclusion that such a property is rather uncommon among dairy LAB [38,66].

Overviewing the results obtained in the present study, many strains, belonging to different species presented very interesting properties. However, these properties were scattered among the strains and no strain would present more than one. More specifically, the strains able to produce antilisterial compounds were poor acidifiers and among the least susceptible to antibiotics. Therefore, selection could only be based on fast acidification capacity among the strains that were more susceptible to antibiotics; these strains were *Lc. lactis* DRD 2692 and *P. pentosaceus* DRD 2655.

## 5. Conclusions

The properties assessed in the present study are strain- rather than species-dependent. It was further supported that proteolysis, lipolysis, EPS and bacteriocin production, amino acid decarboxylation, and hemolytic and gelatinase activity are rather uncommon traits among dairy LAB. However, resistance to antibiotics seems very common. Many strains, belonging to different species, have presented very interesting properties, which deserve further assessment.

## Figures and Tables

**Table 1:** Skim milk acidification (in ΔpH ^1^) and coagulation capacity of representative strains of the LAB included in the present study.

Strain (DRD)	Incubation Time (h)								Coagulation ^2^
	3	6	9	12	15	18	21	24	
* E. faecalis *									
2602	0.08 (0.017)	0.21 (0.011)	0.38 (0.020)	0.50 (0.010)	0.57 (0.036)	0.66 (0.034)	0.74 (0.066)	0.76 (0.047)	24 h
2651	0.06 (0.005)	0.27 (0.026)	0.38 (0.025)	0.54 (0.061)	0.99 (0.065)	1.10 (0.050)	0.94 (0.615)	1.37 (0.028)	24 h
2722	0.08 (0.043)	0.31 (0.057)	0.50 (0.029)	0.64 (0.015)	0.80 (0.025)	0.88 (0.032)	0.95 (0.053)	0.98 (0.040)	24 h
* Lc. lactis *									
2649	0.05 (0.076)	0.28 (0.096)	0.20 (0.049)	1.74 (0.021)	1.92 (0.032)	2.17 (0.006)	2.16 (0.036)	2.13 (0.025)	12 h
2658	0.07 (0.070)	0.61 (0.095)	1.81 (0.025)	2.05 (0.030)	2.22 (0.015)	2.23 (0.006)	2.16 (0.140)	2.27 (0.035)	6 h
2692	0.08 (0.031)	0.47 (0.146)	1.35 (0.311)	1.91 (0.066)	2.11 (0.036)	2.13 (0.043)	2.17 (0.026)	2.18 (0.030)	6 h
2699	0.08 (0.040)	0.12 (0.050)	0.16 (0.032)	0.17 (0.032)	0.11 (0.049)	0.19 (0.01)	0.19 (0.038)	0.21 (0.041)	NC
* Lp. pentosus *									
2662	0.10 (0.043)	0.53 (0.217)	1.38 (0.361)	1.94 (0.085)	2.16 (0.057)	2.16 (0.063)	2.20 (0.028)	2.18 (0.066)	6 h
2693	0.09 (0.034)	0.10 (0.045)	0.14 (0.025)	0.16 (0.030)	0.15 (0.066)	0.19 (0.02)	0.19 (0.023)	0.18 (0.020)	NC
*Lt. curvatus*									
2708	0.03 (0.005)	0.10 (0.015)	0.24 (0.040)	0.31 (0.020)	0.45 (0.010)	0.50 (0.011)	0.54 (0.010)	0.59 (0.023)	24 h
2709	0.05 (0.020)	0.19 (0.026)	0.33 (0.051)	0.48 (0.034)	0.73 (0.055)	0.78 (0.049)	0.85 (0.041)	0.89 (0.050)	3 d
2716	0.03 (0.015)	0.13 (0.020)	0.25 (0.020)	0.34 (0.035)	0.64 (0.058)	0.74 (0.035)	0.89 (0.015)	0.96 (0.030)	2 d
2728	0.04 (0.025)	0.10 (0.025)	0.16 (0.035)	0.20 (0.041)	0.24 (0.017)	0.23 (0.020)	0.24 (0.015)	0.23 (0.047)	NC
* E. faecium *									
2579	0.08 (0.02)	0.24 (0.03)	0.36 (0.037)	0.40 (0.045)	0.50 (0.065)	0.52 (0.070)	0.55 (0.075)	0.58 (0.089)	4 d
2581	0.07 (0.020)	0.19 (0.030)	0.29 (0.036)	0.32 (0.035)	0.39 (0.047)	0.40 (0.020)	0.40 (0.015)	0.43 (0.015)	NC
2585	0.06 (0.023)	0.17 (0.028)	0.29 (0.030)	0.33 (0.045)	0.35 (0.015)	0.47 (0.015)	0.46 (0.020)	0.43 (0.055)	NC
2588	0.07 (0.028)	0.15 (0.030)	0.30 (0.026)	0.34 (0.035)	0.39 (0.052)	0.51 (0.017)	0.48 (0.055)	0.46 (0.061)	4 d
2667	0.05 (0.045)	0.22 (0.025)	0.58 (0.085)	0.91 (0.005)	1.21 (0.023)	1.37 (0.011)	1.46 (0.01)	1.52 (0.026)	12 h
2670	0.07 (0.036)	0.22 (0.036)	0.60 (0.020)	0.85 (0.045)	1.10 (0.025)	1.29 (0.023)	1.37 (0.02)	1.39 (0.026)	24 h
2587	0.05 (0.026)	0.07 (0.037)	0.07 (0.011)	0.07 (0.005)	0.08 (0.030)	0.13 (0.02)	0.12 (0.02)	0.11 (0.075)	NC
2600	0.08 (0.017)	0.24 (0.037)	0.46 (0.025)	0.59 (0.020)	0.64 (0.015)	0.70 (0.015)	0.72 (0.037)	0.72 (0.015)	3 d
2695	0.04 (0.083)	0.08 (0.115)	0.12 (0.110)	0.21 (0.173)	0.21 (0.023)	0.26 (0.025)	0.42 (0.191)	0.42 (0.026)	3 d
2711	0.04 (0.032)	0.12 (0.045)	0.25 (0.055)	0.33 (0.100)	0.53 (0.040)	0.72 (0.03)	0.81 (0.030)	0.83 (0.126)	3 d
2720	0.07 (0.030)	0.19 (0.025)	0.36 (0.026)	0.38 (0.030)	0.49 (0.015)	0.56 (0.017)	0.56 (0.030)	0.55 (0.126)	5 d
2723	0.05 (0.005)	0.21 (0.025)	0.30 (0.026)	0.32 (0.030)	0.44 (0.015)	0.43 (0.017)	0.45 (0.030)	0.51 (0.126)	NC
2691	0.07 (0.030)	0.16 (0.076)	0.49 (0.080)	0.75 (0.076)	1.00 (0.017)	1.23 (0.030)	1.35 (0.005)	1.39 (0.02)	24 h
2686	0.05 (0.001)	0.19 (0.003)	0.27 (0.020)	0.31 (0.015)	0.39 (0.030)	0.36 (0.030)	0.40 (0.030)	0.41 (0.052)	NC
2724	0.07 (0.036)	0.19 (0.010)	0.35 (0.020)	0.44 (0.040)	0.54 (0.037)	0.63 (0.005)	0.68 (0.035)	0.78 (0.181)	6 d
* P. pentosaceus *									
2616	0.1 (0.032)	0.15 (0.005)	0.23 (0.020)	0.29 (0.026)	0.30 (0.02)	0.32 (0.051)	0.38 (0.040)	0.41 (0.040)	NC
2635	0.11 (0.025)	0.20 (0.040)	0.32 (0.026)	0.39 (0.005)	0.48 (0.041)	0.52 (0.023)	0.53 (0.025)	0.54 (0.021)	NC
2681	0.07 (0.066)	0.11 (0.055)	0.21 (0.010)	0.42 (0.051)	0.50 (0.032)	0.77 (0.121)	1.00 (0.115)	1.13 (0.085)	3 d
2689	0.06 (0.02)	0.14 (0.049)	0.32 (0.047)	0.71 (0.060)	1.08 (0.041)	1.30 (0.005)	1.44 (0.041)	1.47 (0.026)	24 h
2694	0.01 (0.017)	0.03 (0.036)	0.06 (0.07)	0.13 (0.090)	0.73 (0.936)	0.83 (0.990)	0.87 (1.013)	0.88 (0.962)	3 d
2753	0.09 (0.062)	0.16 (0.130)	0.21 (0.225)	0.23 (0.239)	0.15 (0.015)	0.18 (0.023)	0.24 (0.032)	0.31 (0.106)	NC
2634	0.08 (0.034)	0.12 (0.045)	0.16 (0.025)	0.17 (0.011)	0.15 (0.068)	0.22 (0.02)	0.22 (0.025)	0.23 (0.001)	NC
2652	0.10 (0.040)	0.30 (0.045)	0.50 (0.055)	0.73 (0.051)	1.10 (0.015)	1.30 (0.001)	1.44 (0.011)	1.49 (0.023)	12 h
2655	0.11 (0.025)	0.58 (0.050)	1.12 (0.015)	1.53 (0.025)	1.78 (0.050)	1.86 (0.025)	1.92 (0.005)	1.96 (0.069)	6 h
2657	0.11 (0.026)	0.66 (0.070)	1.85 (0.020)	2.00 (0.037)	2.21 (0.011)	2.22 (0.020)	2.23 (0.015)	2.22 (0.064)	6 h
2761	0.08 (0.011)	0.18 (0.036)	0.26 (0.043)	0.35 (0.030)	0.42 (0.005)	0.48 (0.001)	0.58 (0.020)	0.65 (0.005)	2 d
2679	0.01 (0.023)	0.06 (0.032)	0.33 (0.020)	0.44 (0.023)	0.01 (0.034)	0.01 (0.017)	0.13 (0.199)	0.18 (0.238)	NC
2676	0.07 (0.061)	0.12 (0.086)	0.22 (0.097)	0.33 (0.070)	0.45 (0.011)	0.63 (0.075)	0.66 (0.126)	0.65 (0.188)	4 d
2748	0.10 (0.015)	0.24 (0.028)	0.34 (0.036)	0.42 (0.020)	0.54 (0.020)	0.60 (0.028)	0.67 (0.02)	0.72 (0.043)	24 h
2749	0.08 (0.005)	0.21 (0.02)	0.29 (0.032)	0.4 (0.043)	0.52 (0.028)	0.58 (0.036)	0.72 (0.005)	0.89 (0.026)	2 d
2752	0.09 (0.011)	0.18 (0.020)	0.25 (0.030)	0.30 (0.010)	0.31 (0.011)	0.31 (0.015)	0.36 (0.026)	0.42 (0.030)	6 d
* Ln. mesenteroides *									
2576	0.09 (0.020)	0.22 (0.011)	0.32 (0.015)	0.36 (0.020)	0.40 (0.011)	0.42 (0.005)	0.39 (0.030)	0.38 (0.01)	NC
2604	0.12 (0.02)	0.43 (0.026)	0.93 (0.026)	1.29 (0.055)	1.50 (0.075)	1.65 (0.060)	1.72 (0.034)	1.74 (0.060)	12 h
2703	0.06 (0.080)	0.18 (0.189)	0.56 (0.258)	0.88 (0.150)	1.08 (0.085)	1.33 (0.086)	1.42 (0.075)	1.44 (0.051)	NC
2705	0.07 (0.055)	0.12 (0.160)	0.32 (0.149)	0.39 (0.112)	0.47 (0.041)	0.60 (0.030)	0.62 (0.040)	0.66 (0.092)	4 d
2706	0.01 (0.017)	0.02 (0.051)	0.03 (0.058)	0.10 (0.159)	0.32 (0.375)	0.51 (0.540)	0.74 (0.564)	0.64 (0.242)	24 h
* Lp. plantarum *									
2584	0.07 (0.032)	0.19 (0.041)	0.34 (0.015)	0.42 (0.032)	0.55 (0.02)	0.64 (0.015)	0.66 (0.005)	0.66 (0.02)	3 d
2608	0.14 (0.005)	0.30 (0.032)	0.51 (0.051)	0.64 (0.025)	0.85 (0.130)	0.91 (0.145)	0.99 (0.137)	1.05 (0.151)	2 d
2653	0.10 (0.028)	0.30 (0.026)	0.50 (0.026)	0.78 (0.017)	1.05 (0.101)	1.25 (0.092)	1.39 (0.083)	1.48 (0.085)	12 h
2668	0.07 (0.043)	0.06 (0.050)	0.07 (0.032)	0.37 (0.589)	0.60 (0.335)	1.32 (0.526)	2.00 (0.104)	2.05 (0.068)	24 h
2612	0.10 (0.020)	0.23 (0.055)	0.37 (0.098)	0.50 (0.134)	0.61 (0.185)	0.67 (0.189)	0.73 (0.172)	0.77 (0.163)	2 d
2645	0.05 (0.005)	0.18 (0.037)	0.29 (0.028)	0.31 (0.015)	0.44 (0.025)	0.44 (0.025)	0.46 (0.026)	0.47 (0.025)	4 d
2648	0.06 (0.032)	0.14 (0.045)	0.24 (0.05)	0.43 (0.060)	0.90 (0.066)	1.19 (0.040)	1.31 (0.041)	1.36 (0.051)	24 h
2656	0.08 (0.015)	0.23 (0.005)	0.41 (0.020)	0.52 (0.126)	0.42 (0.040)	0.47 (0.030)	0.47 (0.040)	0.51 (0.037)	3 d
* Lv. brevis *									
2596	0.05 (0.015)	0.11 (0.017)	0.24 (0.01)	0.33 (0.047)	0.67 (0.075)	0.77 (0.051)	0.88 (0.026)	0.95 (0.015)	2 d
2624	0.07 (0.005)	0.12 (0.005)	0.16 (0.005)	0.19 (0.015)	0.15 (0.047)	0.21 (0.069)	0.21 (0.005)	0.56 (0.597)	NC
2664	0.05 (0.047)	0.12 (0.205)	0.30 (0.090)	1.63 (0.272)	1.98 (0.165)	2.14 (0.120)	2.20 (0.075)	2.16 (0.066)	6 h
2665	0.06 (0.055)	0.22 (0.015)	0.39 (0.052)	0.44 (0.015)	0.45 (0.015)	0.46 (0.037)	0.48 (0.017)	0.56 (0.085)	3 d
2757	0.09 (0.040)	0.20 (0.020)	0.23 (0.005)	0.28 (0.005)	0.28 (0.03)	0.29 (0.017)	0.31 (0.032)	0.35 (0.017)	NC
2760	0.06 (0.005)	0.12 (0.005)	0.15 (0.005)	0.18 (0.015)	0.15 (0.005)	0.17 (0.005)	0.23 (0.011)	0.25 (0.015)	NC
2763	0.06 (0.01)	0.12 (0.01)	0.14 (0.01)	0.19 (0.015)	0.13 (0.015)	0.15 (0.015)	0.21 (0.023)	0.24 (0.040)	NC
2688	0.1 (0.02)	0.13 (0.051)	0.16 (0.036)	0.18 (0.040)	0.09 (0.058)	0.25 (0.046)	0.26 (0.017)	0.26 (0.034)	NC
* W. paramesenteroides *									
2613	0.11 (0.028)	0.20 (0.010)	0.36 (0.010)	0.43 (0.015)	0.51 (0.020)	0.53 (0.005)	0.60 (0.075)	0.61 (0.062)	NC
2701	0.10 (0.040)	0.13 (0.055)	0.17 (0.041)	0.19 (0.041)	0.14 (0.050)	0.18 (0.052)	0.21 (0.020)	0.21 (0.026)	NC
2714	0.10 (0.030)	0.19 (0.01)	0.19 (0.107)	0.36 (0.028)	0.50 (0.017)	0.56 (0.020)	0.54 (0.028)	0.55 (0.010)	NC
2719	0.03 (0.010)	0.10 (0.011)	0.25 (0.047)	0.57 (0.118)	1.09 (0.049)	1.19 (0.035)	1.33 (0.026)	1.40 (0.035)	24 h
2751	0.07 (0.005)	0.19 (0.010)	0.29 (0.010)	0.37 (0.017)	0.55 (0.020)	0.57 (0.025)	0.61 (0.038)	0.62 (0.040)	4 d
2726	0.09 (0.032)	0.20 (0.040)	0.32 (0.015)	0.42 (0.011)	0.52 (0.020)	0.54 (0.025)	0.57 (0.025)	0.64 (0.023)	4 d
2743	0.08 (0.041)	0.24 (0.037)	0.66 (0.036)	0.90 (0.049)	1.06 (0.050)	1.19 (0.005)	1.26 (0.020)	1.33 (0.005)	24 h
2755	0.10 (0.023)	0.20 (0.020)	0.30 (0.011)	0.39 (0.034)	0.53 (0.026)	0.53 (0.020)	0.55 (0.015)	0.56 (0.025)	NC
2756	0.07 (0.026)	0.25 (0.025)	0.39 (0.005)	0.48 (0.015)	0.61 (0.011)	0.62 (0.015)	0.65 (0.036)	0.66 (0.015)	3 d

^1^ The ΔpH values were calculated by subtracting the pH value after the given incubation time at 30 °C (37 °C for enterococci) from the respective of uninoculated skim milk incubated for the same time at the same temperature. ^2^ The time in which coagulation was visibly observed in presented. NC: no coagulation after 7 days incubation at 37 °C for enterococci and 30 °C for the rest LAB.

## Data Availability

The data presented in this study are available in the manuscript.

## Supplementary Materials

The following supporting information can be downloaded at: https://www.mdpi.com/article/10.3390/foods11030459/s1, Figure S1: Microbial survival (in log N/N_0_) after exposure of *E. faecalis* (**A**), *E. faecium* (**B**), *Lc. lactis* (**C**), *Ln. mesenteroides* (**D**), *Lp. pentosus* (**E**), *Lp. plantarum* (**F**), *Lt. curvatus* (**G**), *Lv. brevis* (**H**), *P. pentosaceus* (**I**) and *W. paramesenteroides* (**J**) strains included in the present study to PBS buffer adjusted to pH 2 (blue) or 3 (green) with HCl and incubation at 37 °C for 3 h. N: microbial population after incubation; N_0_: initial microbial population. Figure S2: Microbial survival (in log N/N_0_) after exposure of *E. faecalis* (**A**), *E. faecium* (**B**), *Lc. lactis* (**C**), *Ln. mesenteroides* (**D**), *Lp. pentosus* (**E**), *Lp. plantarum* (**F**), *Lt. curvatus* (**G**), *Lv. brevis* (**H**), *P. pentosaceus* (**I**) and *W. paramesenteroides* (**J**) strains included in the present study to PBS buffer, pH 8, containing 0.5% (blue), 1.0% (green) or 2.0% (yellow) (*w*/*v*) bile bovine and incubation at 37 °C for 4 h. N: microbial population after incubation; N_0_: initial microbial population. Table S1: Origin of the strains included in the present study.
