# Supplementary material for: Microbial Ecology of Sheep Milk, Artisanal Feta, and Kefalograviera Cheeses. Part II: Technological, Safety, and Probiotic Attributes of Lactic Acid Bacteria Isolates
Source: Foods. 2022 Feb 3;11(3):459. doi: 10.3390/foods11030459 (PMC8834287; doi:10.3390/foods11030459)
Supplement: Supplementary file 1 [file foods-11-00459-s001.zip › foods-1536024-supplementary/Figures S1 & S2_revised.pdf]

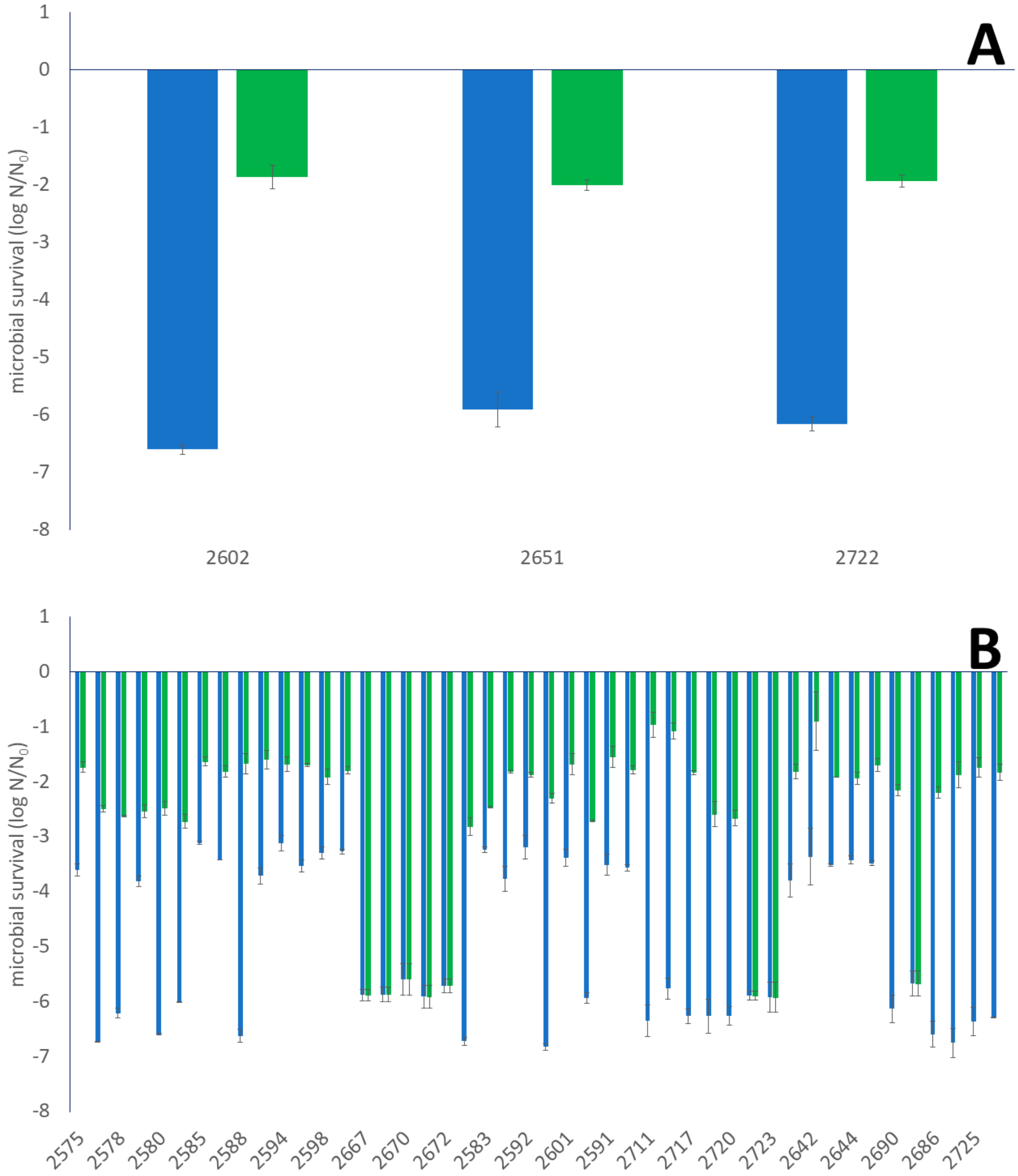
A
microbial survival (log N/N_0)
1
0
-1
-2
-3
-4
-5
-6
-7
-8
2602
2651
2722
B
microbial survival (log N/N_0)
1
0
-1
-2
-3
-4
-5
-6
-7
-8
2575
2578
2580
2585
2588
2594
2598
2667
2670
2672
2583
2592
2601
2591
2711
2717
2720
2723
2642
2644
2690
2686
2725

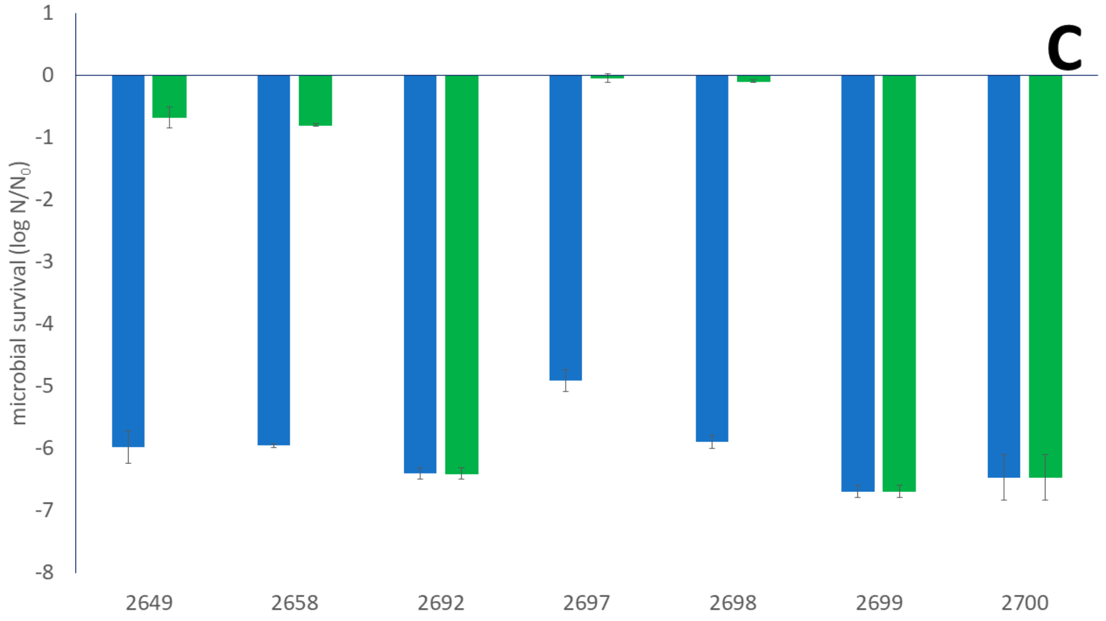
C
microbial survival (log N/N_0)
1
0
-1
-2
-3
-4
-5
-6
-7
-8
2649
2658
2692
2697
2698
2699
2700

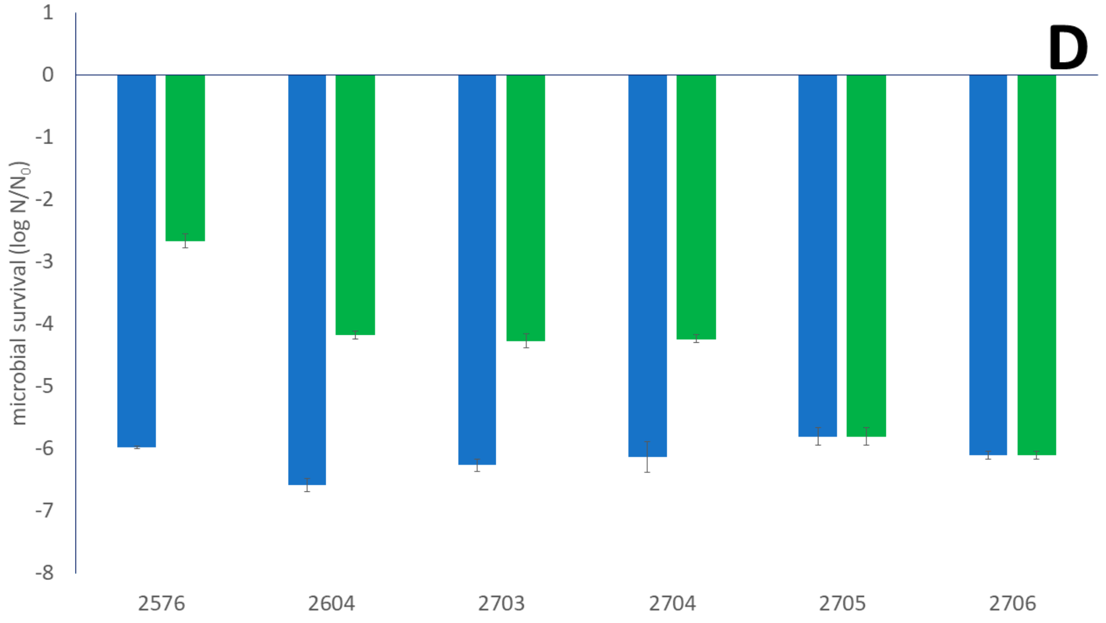
D
microbial survival (log N/N_0)
1
0
-1
-2
-3
-4
-5
-6
-7
-8
2576
2604
2703
2704
2705
2706

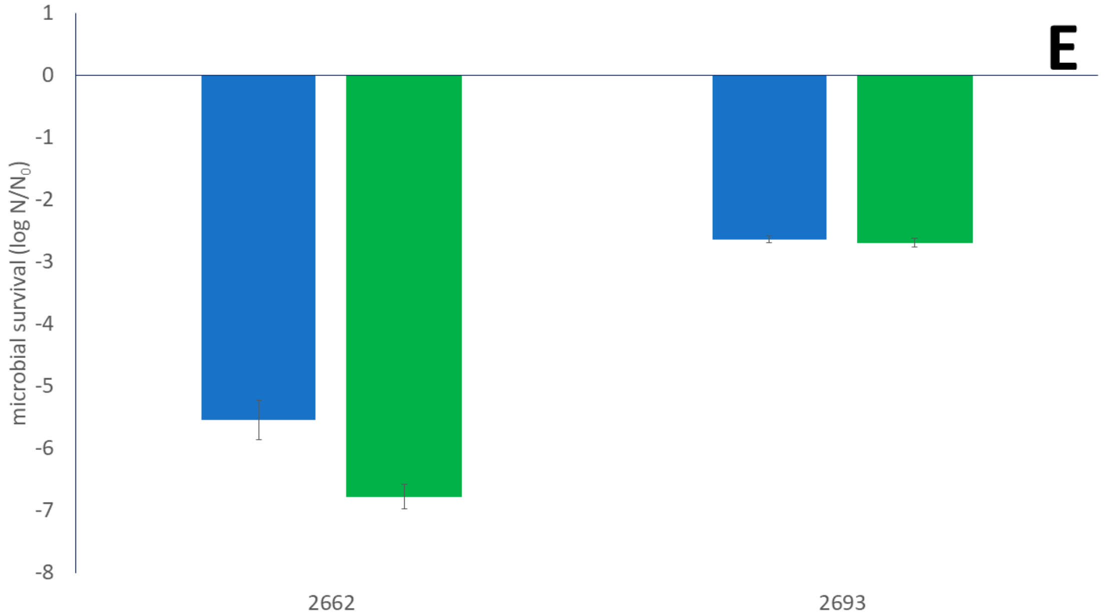
E
microbial survival (log N/N0)
1
0
-1
-2
-3
-4
-5
-6
-7
-8
2662
2693

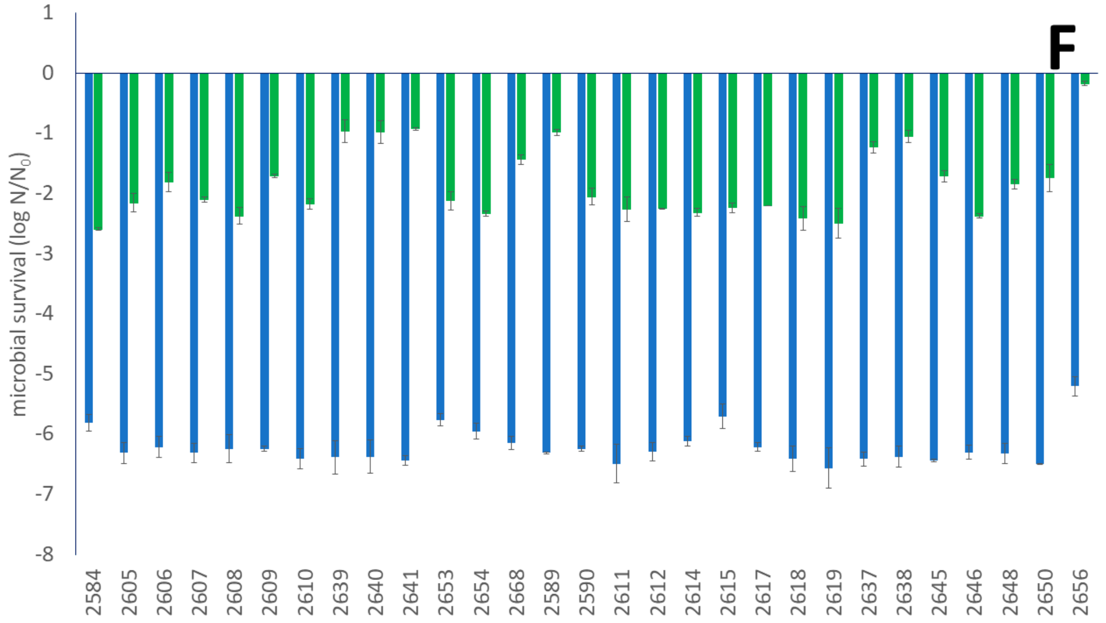
F
microbial survival (log N/N0)
1
0
-1
-2
-3
-4
-5
-6
-7
-8
2584
2605
2606
2607
2608
2609
2610
2639
2640
2641
2653
2654
2668
2589
2590
2611
2612
2614
2615
2617
2618
2619
2637
2638
2645
2646
2648
2650
2656

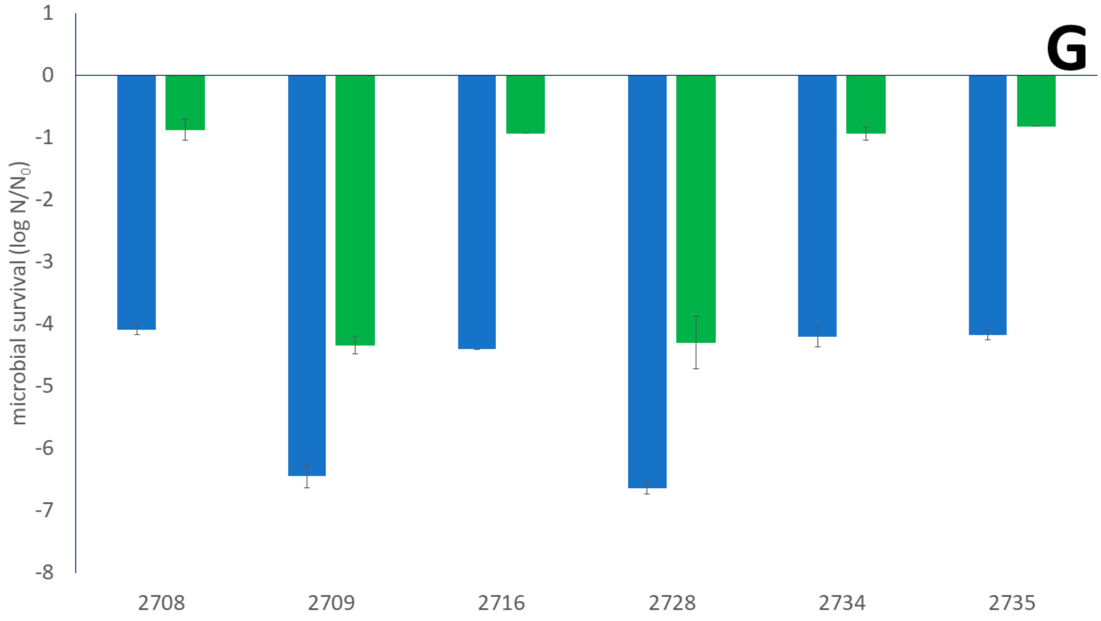
G
microbial survival (log N/N0)
1
0
-1
-2
-3
-4
-5
-6
-7
-8
2708
2709
2716
2728
2734
2735

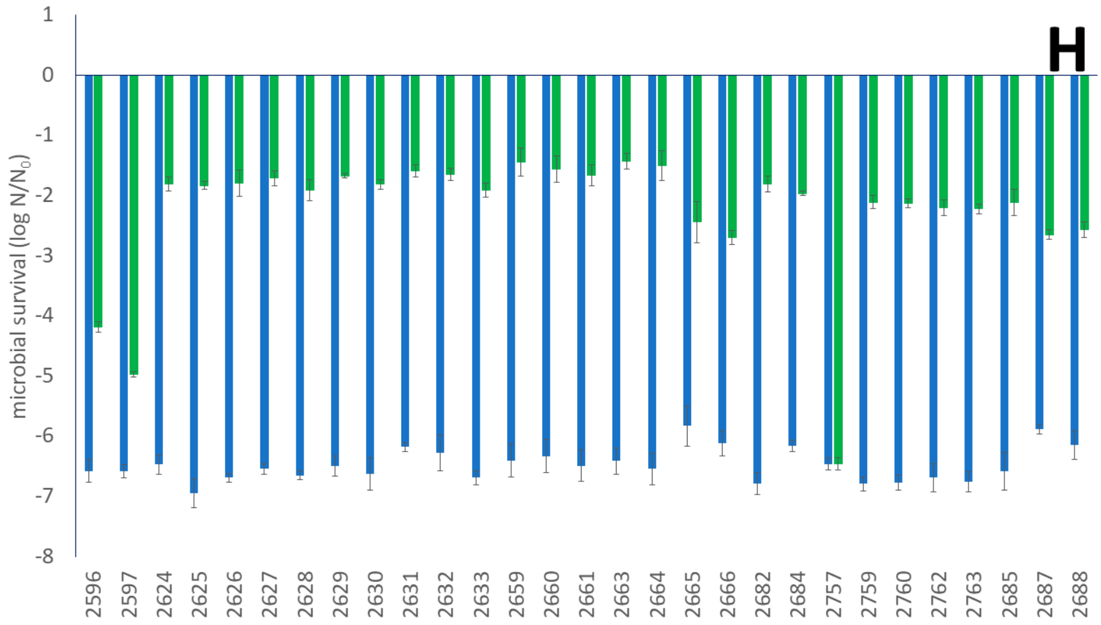
H
microbial survival (log N/N0)
1
0
-1
-2
-3
-4
-5
-6
-7
-8
2596
2597
2624
2625
2626
2627
2628
2629
2630
2631
2632
2633
2659
2660
2661
2663
2664
2665
2666
2682
2684
2757
2759
2760
2762
2763
2685
2687
2688

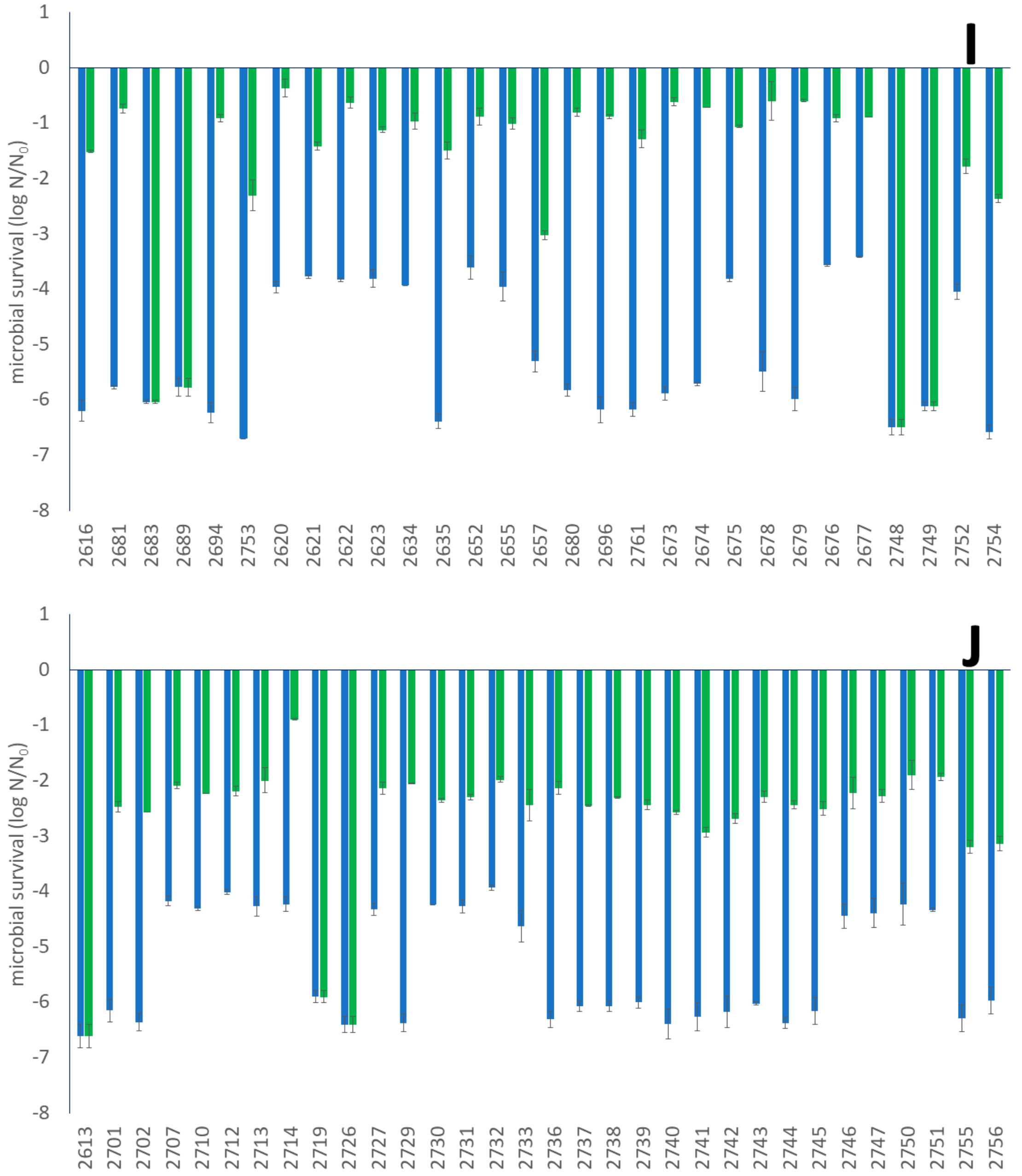

Figure S1. Microbial survival (in log N/N_0) after exposure of *E. faecalis* (**A**), *E. faecium* (**B**), *Lc. lactis* (**C**), Ln. *mesenteroides* (**D**), *Lp. pentosus* (**E**), *Lp. plantarum* (**F**), *Lt. curvatus* (**G**), *Lv. brevis* (**H**), *P. pentosaceus* (**I**) and *W. paramesenteroides* (**J**) strains included in the present study to PBS buffer adjusted to pH 2 (blue) or 3 (green) with HCl and incubation at 37 ºC for 3 h. N: microbial population after incubation; N_0: initial microbial population.

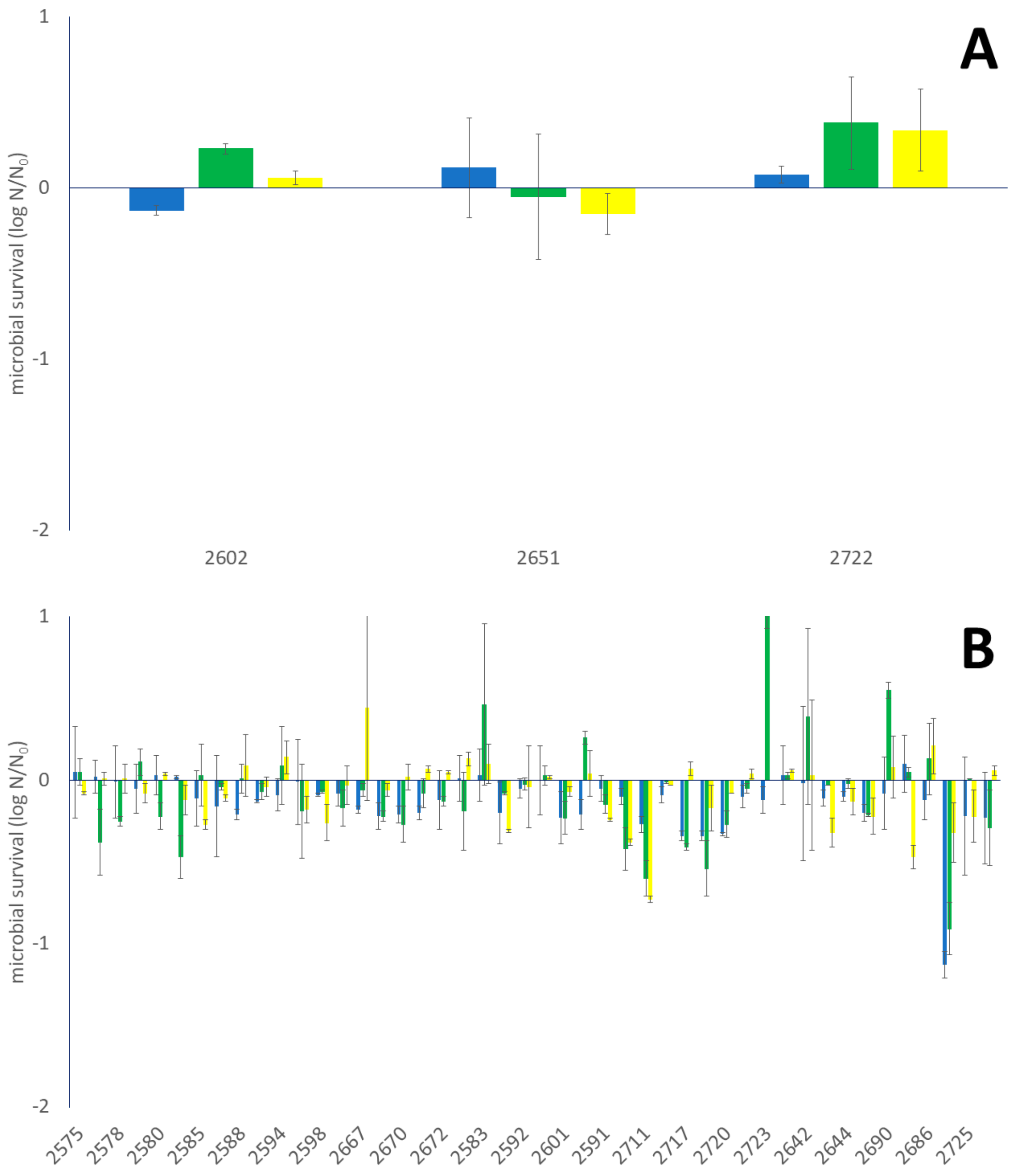
A
B
1
0
-1
-2
microbial survival (log N/N0)
2602
2651
2722
2575
2578
2580
2585
2588
2594
2598
2667
2670
2672
2583
2592
2601
2591
2711
2717
2720
2723
2642
2644
2690
2686
2725

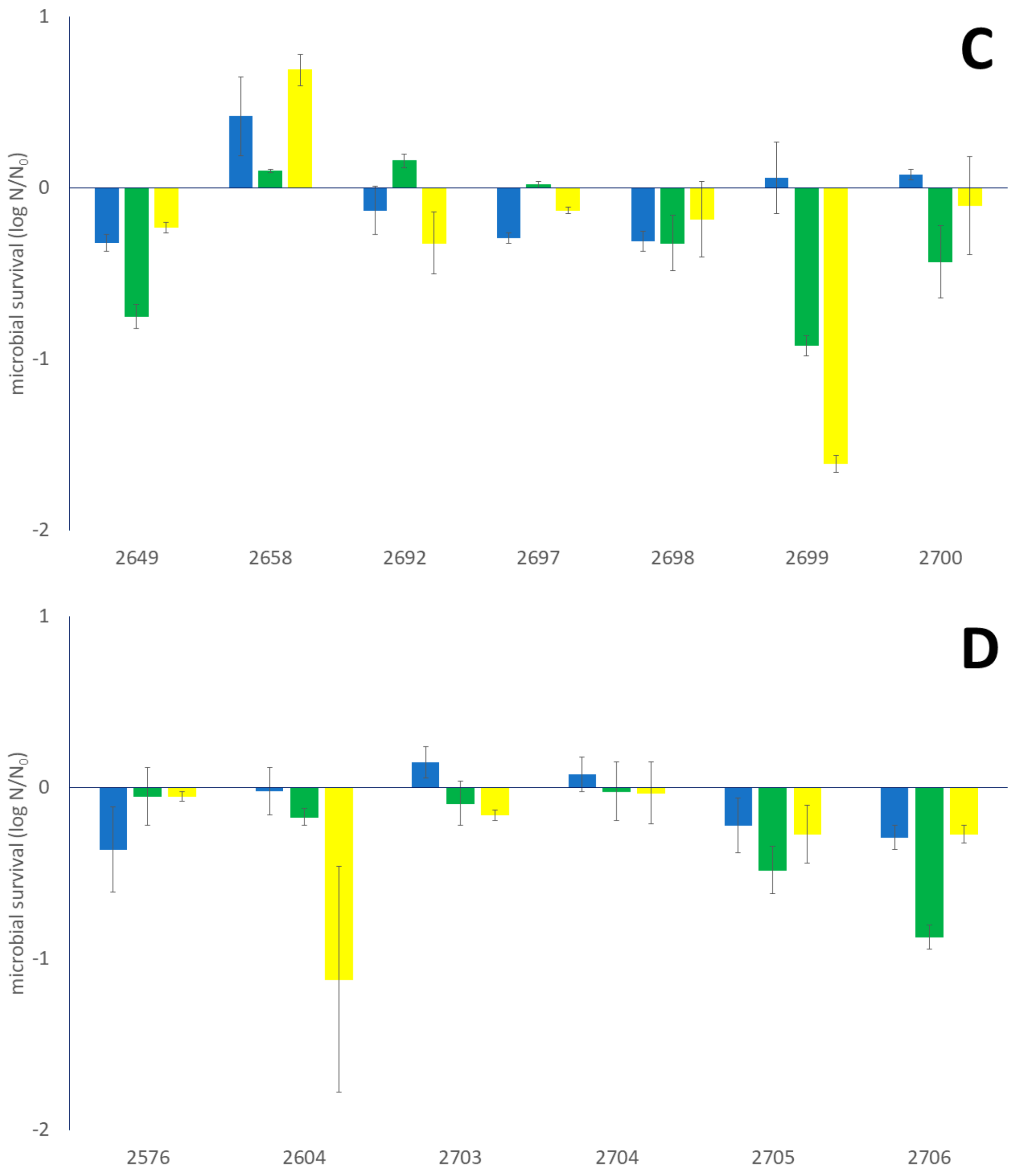
C
microbial survival (log N/N0)
1
0
-1
-2
2649
2658
2692
2697
2698
2699
2700
D
microbial survival (log N/N0)
1
0
-1
-2
2576
2604
2703
2704
2705
2706

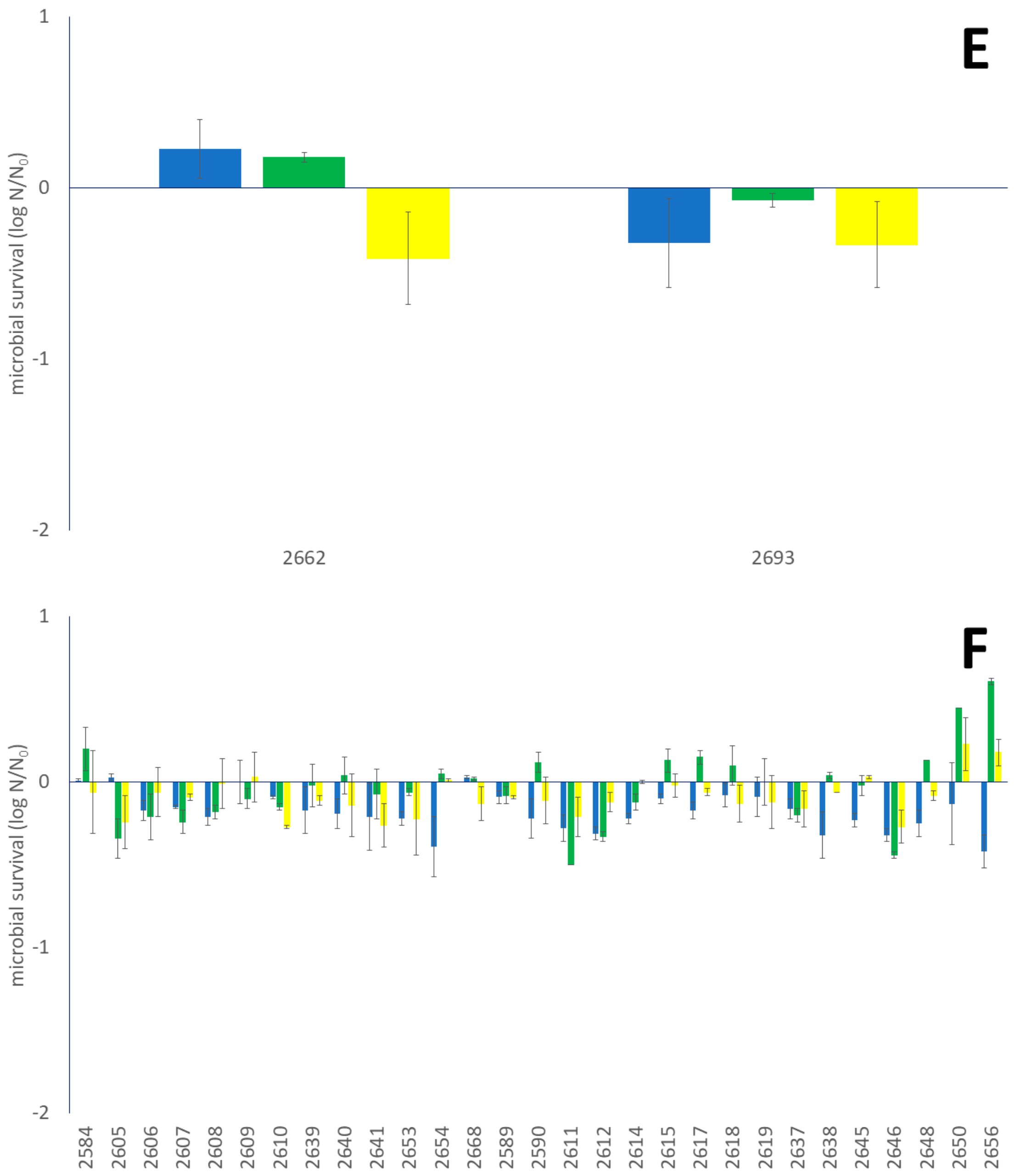
E
F
microbial survival (log N/N0)
1
0
-1
-2
2662
2693
2584
2605
2606
2607
2608
2609
2610
2639
2640
2641
2653
2654
2668
2589
2590
2611
2612
2614
2615
2617
2618
2619
2637
2638
2645
2646
2648
2650
2656

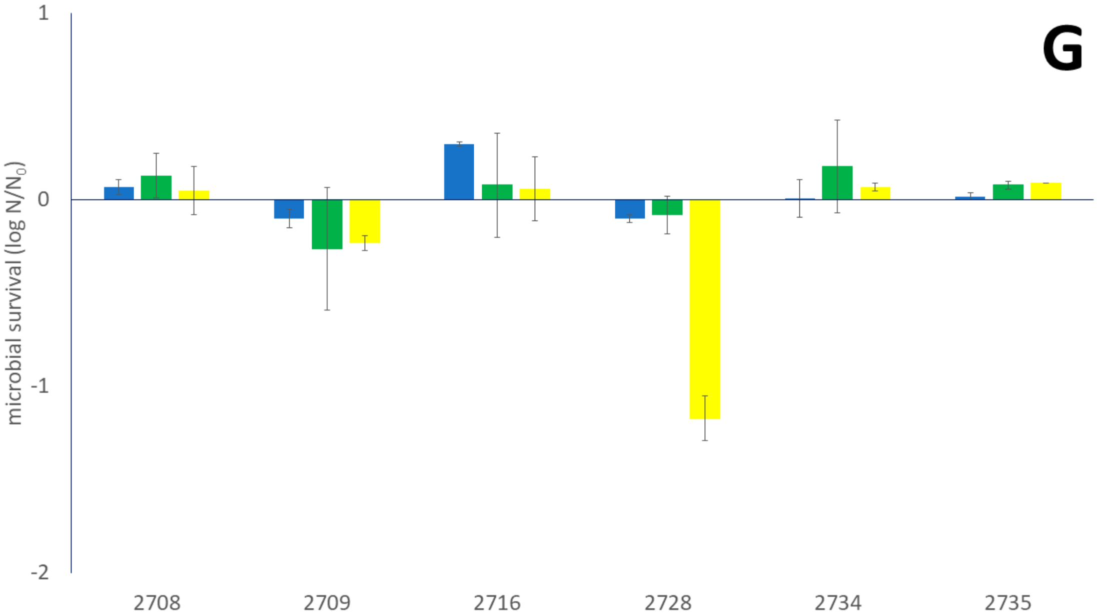
G
microbial survival (log N/N0)
1
0
-1
-2
2708
2709
2716
2728
2734
2735

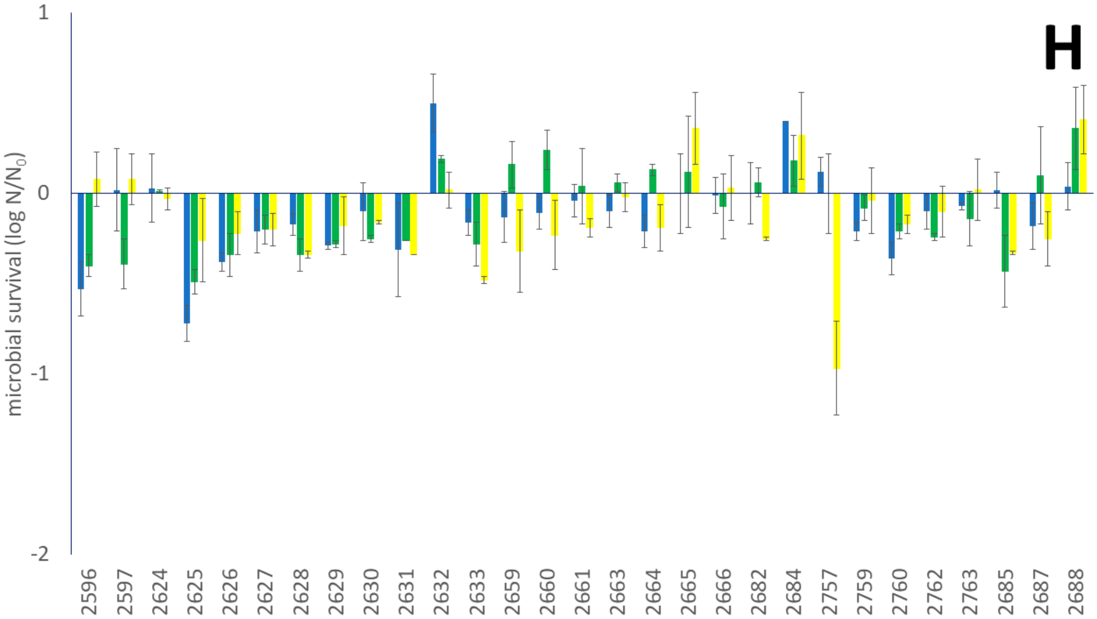
H
microbial survival (log N/N0)
1
0
-1
-2
2596
2597
2624
2625
2626
2627
2628
2629
2630
2631
2632
2633
2659
2660
2661
2663
2664
2665
2666
2682
2684
2757
2759
2760
2762
2763
2685
2687
2688

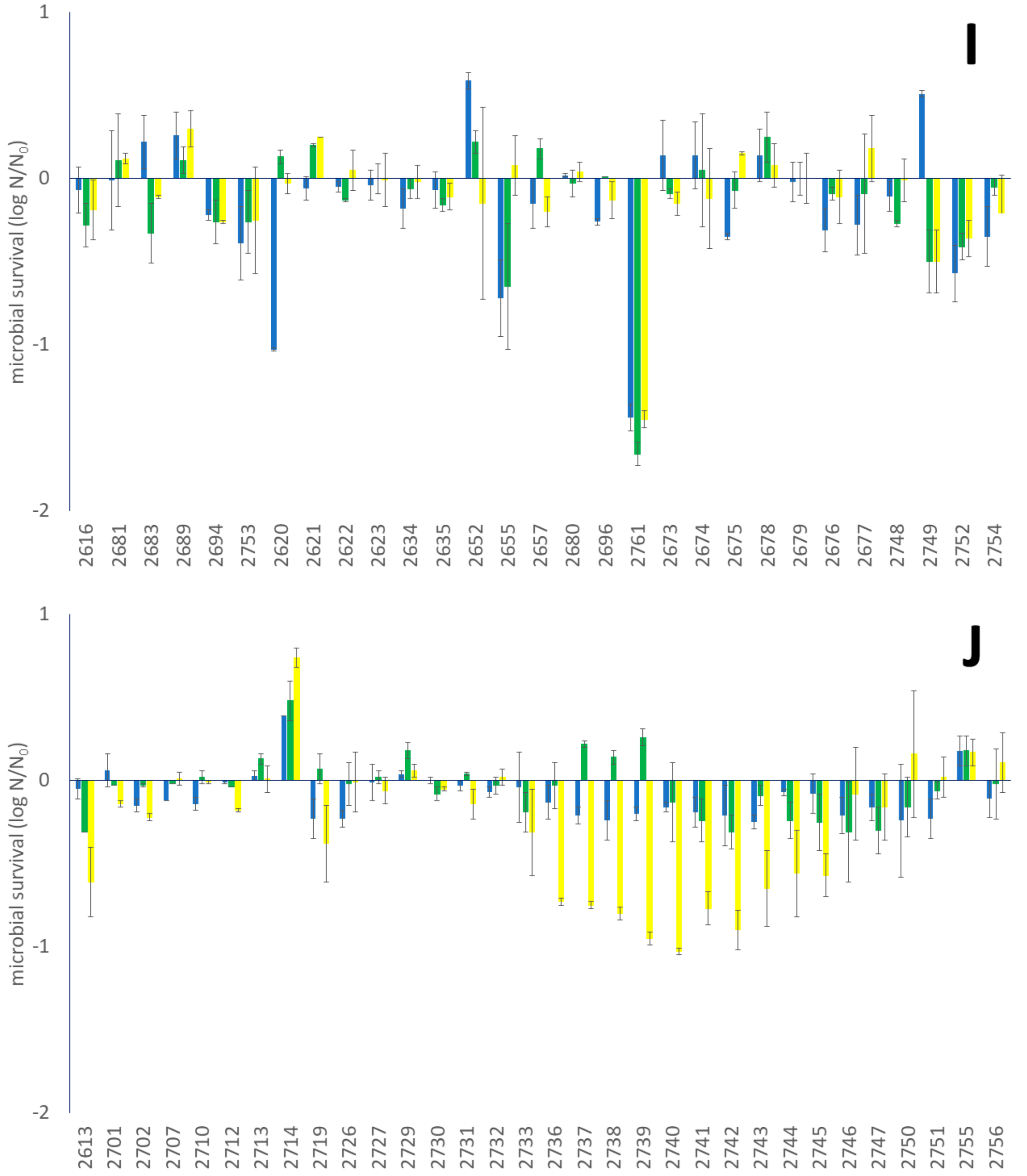

Figure S2. Microbial survival (in log N/N_0) after exposure of *E. faecalis* (**A**), *E. faecium* (**B**), *Lc. lactis* (**C**), Ln. *mesenteroides* (**D**), *Lp. pentosus* (**E**), *Lp. plantarum* (**F**), *Lt. curvatus* (**G**), *Lv. brevis* (**H**), *P. pentosaceus* (**I**) and *W. paramesenteroides* (**J**) strains included in the present study to PBS buffer, pH 8, containing 0.5% (blue), 1.0% (green) or 2.0% (yellow) (*w*/*v*) bile bovine and incubation at 37 °C for 4 h. N: microbial population after incubation; N_0: initial microbial population.
