# Supplementary material for: Microbial Ecology of Sheep Milk, Artisanal Feta, and Kefalograviera Cheeses. Part II: Technological, Safety, and Probiotic Attributes of Lactic Acid Bacteria Isolates
Source: Foods. 2022 Feb 3;11(3):459. doi: 10.3390/foods11030459 (PMC8834287; doi:10.3390/foods11030459)
Supplement: Supplementary file 1 [file foods-11-00459-s001.zip › foods-1536024-supplementary/Table S1_revised.pdf]

Table S1. Origin of the strains included in the present study.

Species	Origin				
	Sheep Milk	Feta		Kefalograviera	
		Early Ripening	Late Ripening	Early Ripening	Late Ripening
E. faecium	2647, 2667, 2669, 2670, 2671, 2672, 2686, 2690, 2691, 2695	2585, 2586, 2587, 2588, 2591, 2592, 2593, 2594, 2595, 2598, 2599, 2600, 2601, 2603, 2642, 2643, 2644	2711, 2715	2757, 2577, 2578, 2579, 2580, 2581, 2582, 2583, 2636	2717, 2718, 2720, 2721, 2723, 2724, 2725, 2758
E. faecalis	2651	2602			2722
Lc. lactis	2649, 2658, 2692, 2697, 2698, 2699, 2700				
Ln. mesenteroides	2703, 2704, 2705, 2706	2604		2576	
Lp. pentosus	2662, 2693				
Lp. plantarum	2648, 2650, 2653, 2654, 2656, 2668	2589, 2590, 2605, 2606, 2607, 2608, 2609, 2610, 2611, 2612, 2614, 2615, 2617, 2618, 2619, 2637, 2638, 2639, 2640, 2641, 2645, 2646		2584	
Lt. curvatus			2708, 2709, 2716, 2728, 2734, 2735		
Lv. brevis	2659, 2660, 2661, 2663, 2664, 2665,	2596, 2597		2624, 2625, 2626, 2627, 2628, 2629,	2757, 2759, 2760, 2762, 2763

	2666, 2682, 2684, 2685, 2687, 2688			2630, 2631, 2632, 2633	
P. pentosaceus	2652, 2655, 2657, 2673, 2674, 2675, 2676, 2677, 2678, 2679, 2680, 2681, 2683, 2689, 2694, 2696	2616	2748, 2749, 2752, 2753, 2754	2620, 2621, 2622, 2623, 2634, 2635	2761
W. paramesenteroides	2701, 2702	2613	2707, 2710, 2712, 2713, 2714, 2726, 2727, 2729, 2730, 2731, 2732, 2733, 2746, 2747, 2750, 2751, 2755		2719, 2736, 2737, 2738, 2739, 2740, 2741, 2742, 2743, 2744, 2745, 2756

E.: *Enterococcus*; *Lc.*: *Lactococcus*; *Ln.*: *Leuconostoc*; *Lp.*: *Lactiplantibacillus*; *Lt.*: *Latilactobacillus*; *Lv.*: *Levilactobacillus*; *P.*: *Pediococcus*; *W.*: *Weissella.*
